# Differences in constitutive gene expression of cytochrome P450 enzymes and ATP-binding cassette transporter gene expression between a susceptible and a highly macrocyclic lactone-resistant *Haemonchus contortus* isolate in the absence of drug-inducible expression

**DOI:** 10.1186/s13071-024-06568-z

**Published:** 2024-12-12

**Authors:** Natalie Jakobs, Sandro Andreotti, Sabrina Ramünke, Georg von Samson-Himmelstjerna, Jürgen Krücken

**Affiliations:** 1 Institute for Parasitology and Tropical Veterinary Medicine, Freie Universität Berlin, Berlin, Germany; 2 Veterinary Centre for Resistance Research, Freie Universität Berlin, Berlin, Germany; 3 Institute of Computer Science, Bioinformatics Solution Center, Freie Universität Berlin, Berlin, Germany

**Keywords:** *Haemonchus contortus*, Anthelmintic resistance, Cytochrome P450, P-Glycoproteins, ATP-binding cassette transporter, Macrocyclic lactones, Metabolism, Gene expression, RNA sequencing

## Abstract

**Background:**

Anthelmintic resistance in ruminants is a widespread problem that has a severe impact on productivity and animal welfare. The helminth *Haemonchus contortus* is generally considered the most important parasite in small ruminants due to its high pathogenicity and the widespread occurrence of anthelmintic resistance in it. Although the molecular mechanisms associated with resistance against the anthelmintics benzimidazoles (BZs) and levamisole are relatively well understood, the resistance mechanisms against the widely used anthelmintic macrocyclic lactones (MLs) ivermectin (IVM) and moxidectin (MOX) remain poorly understood. Detoxifying enzymes and xenobiotic transporters have been frequently proposed to play a role in ML resistance in multiple organisms, including nematodes.

**Methods:**

The reference genome of *H. contortus* was screened for cytochrome P450 genes (*cyp* genes) by using the Basic Local Alignment Search Tool, and maximum-likelihood phylogenetic analysis was used to assign the sequences to gene families. Fourth-stage larvae of the susceptible (McMaster) and the ML-resistant (Berlin-selected) *H. contortus* isolates were generated in vitro and compared regarding basal expression levels of *cyp* genes and ATP-binding cassette (ABC) transporters by using RNA sequencing. The resistant isolate was further incubated with 100 nM IVM or MOX for 3, 6 and 12 h, and the effects of incubation time and drugs were evaluated.

**Results:**

Twenty-five *cyp* genes were identified in the *H. contortus* genome and assigned to 13 different families. The ML-resistant isolate showed significantly higher and lower constitutive expression of 13 and four *cyp* genes, respectively. Out of the 50 ABC transporter genes, only six showed significantly higher expression in the ML-resistant isolate, while 12 showed lower expression. The fold changes were in general low (range 0.44–5.16). Only *pgp-13* showed significant downregulation in response to IVM (0.77 fold change at 6 h, 0.96 fold change at 12 h) and MOX (0.84 fold change at 12 h). In contrast, *mrp-5* was significantly, albeit minimally, upregulated in the presence of IVM, but not MOX, after 12 h (1.02 fold change).

**Conclusions:**

Despite little observable ML-inducible gene expression in the isolate examined here, some of the changes in the baseline expression levels might well contribute to ML resistance in the context of additional changes in a multigenic resistance model. However, neither *cyp* genes nor the ABC transporters appear to be the main drivers that can explain the high levels of resistance observed in the resistant isolate examined here.

**Graphical Abstract:**

**Figure:**
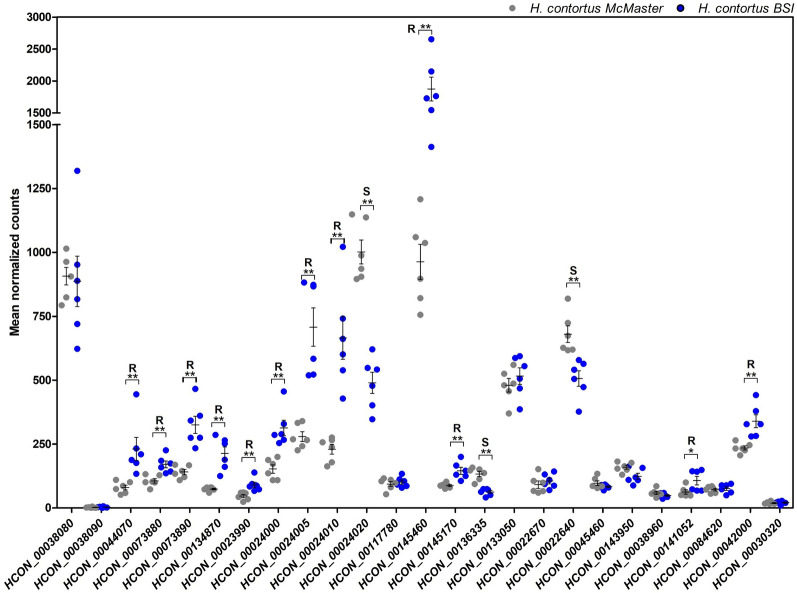

**Supplementary Information:**

The online version contains supplementary material available at 10.1186/s13071-024-06568-z.

## Background

The blood-feeding behavior of the parasitic nematode *Haemonchus contortus*, also known as barber's pole worm, represents a major threat to the livestock industry, and particularly small ruminant production [1]. Although infections with *H. contortus* can be treated with various anthelmintics, such as imidazothiazoles, tetrahydropyrimidines, benzimidazoles (BZs), or macrocyclic lactones (MLs) and amino acetonitrile derivatives, resistance to all classes of drugs is increasing worldwide [2–7]. To manage infections, and limit reinfections, with this highly pathogenic abomasal parasite, there is an urgent need to understand its underlying resistance mechanisms [8]. At present, the mechanisms of anthelmintic resistance, particularly those mediating resistance to MLs, are not fully understood. There is increasing evidence of multigenic mechanisms contributing to ML resistance, such as changes in gene expression of transcription factors [9], drug efflux [10–13], and detoxification via xenobiotic-metabolizing enzymes such as those of the cytochrome P450 family (CYPs) [10, 14] or UDP-glycosyltransferases [15].

Cytochrome P450 enzymes play a pivotal role in catalyzing the oxidative metabolism of xenobiotics, including 70–80% of all those in clinical use [16]. CYPs catalyze various reactions, including N- and O-dealkylation, aliphatic and aromatic hydroxylation, deamination, and N- and S-oxidation [17, 18]. The CYP superfamily's large substrate diversity originates from extensive gene duplications, conversions, gene loss, lateral transfers, and genome duplications [16, 19]. While the human genome encodes 57 putative functional CYPs and 58 pseudogenes, the genome of the nematode *Caenorhabditis* *elegans* encodes 80 CYPs, whose functions are largely unknown [20–22].

The most abundantly expressed CYP in mammals, CYP3A4, substantially contributes to the first-pass metabolism of orally administered drugs [23]. Studies utilizing mammalian liver microsomes to investigate ML metabolite formation revealed that CYP3A4 is primarily responsible for the metabolism of ivermectin (IVM) and moxidectin (MOX) [24–27]. Indeed, an in vitro study using adult *H. contortus* confirmed the role of parasitic nematode CYPs in MOX metabolism, supporting their impact on ML resistance [28]. The upregulation and inducibility of *cyp* gene expression in nematodes upon drug exposure have been revealed in numerous studies, including those on *C. elegans* [21, 22, 29–32], *Teladorsagia circumcincta* [33], *Parascaris univalens* [34], and *H. contortus* [10, 14, 35–37]. Also, the participation of CYPs in ML resistance in *Cooperia oncophora* and *Ostertagia ostertagi* has been supported by data from Al Gusbi et al. [38].

Interestingly, despite IVM and MOX sharing a common ML structure and a similar mode of action due to binding to glutamate-gated chloride channels, there is consistent evidence that both drugs significantly differ in their pharmacokinetics and dynamics in the emergence of resistance [39]. Different studies have proposed that MOX selects less strongly for resistance than IVM [40–42]. Regarding *H. contortus* CYPs ML-specificity, the transgenic expression of the *H. contortus cyp-13A11* in *C. elegans* and exposure to different MLs revealed decreased susceptibility towards IVM but not towards MOX [36]. It remains unclear to what extent and which *H. contortus* CYP family members show IVM- and MOX-specific inducibility.

In addition to biotransformation enzymes such as CYPs, members of the ATP-binding cassette (ABC) protein superfamily also regulate cellular levels of xenobiotics and other small molecules by transporting molecules across cell membranes and epithelia, and hence contribute to detoxification and drug resistance [43].

In mammals, active efflux of structurally unrelated drugs has been associated with ABC transporter subfamilies ABCB1 [P-glycoproteins (PGPs), multidrug resistance-associated protein 1 (MDR1)], ABCC1 [multidrug-resistance-associated protein (MRP1)], ABCC2 (MRP2), ABCC3 (MRP3) and ABCG2 [breast cancer resistance protein (BRCP)] [44–46]. The ability of mammalian PGPs to interact with MLs was demonstrated by Lespine et al. [47].

ABC transporters have also been implicated in ML resistance in several nematodes [48], including *Caenorhabditis elegans* [49–51], cyathostomins [52], *T. circumcincta* [33, 53], *Dirofilaria immitis* [54], *Cyclicocylus elongatus* [55], *Cooperia oncophora* [56, 57] and *P. univalens* [58, 59]. In particular, alterations in PGP expression by individual PGP deletion, silencing, or inhibition were associated with increased IVM susceptibility [29, 49, 50, 60]. Indeed, PGPs were also shown to play a role in *H. contortus* ML resistance mechanisms [37], with studies suggesting a potential impact of *Hco-pgp-2* [11, 61], *Hco-pgp-3* [62], *Hco-pgp-9.1* [11, 63], *Hco-pgp-9.2* [10, 11, 62], *Hco-pgp-10* [10], *Hco-pgp-10* [10], *Hco-pgp-11* [10, 64], and *Hco-pgp-13* [12].

While Williamson et al. [11] demonstrated an increased expression of *Hco-pgp-2* and *Hco-pgp-9* mRNA in a triple-resistant (BZs, levamisole, and IVM) *H. contortus* field isolate, Mate et al. [62] showed differential gene expression of *Hco-pgp-9.2* in IVM-resistant compared to susceptible *H. contortus* adults. The inducibility of *Hco-pgp-9.2* expression in response to IVM was also observed by Kellerova et al. [10], who investigated gene expression in a susceptible *H. contortus* isolate 4, 12, and 24 h after IVM exposure.

Most of the studies that have analyzed the role of ABC transporters in ML resistance have focused on IVM. However, several studies indicated differences in the interaction of IVM and MOX with ABC transporters. The ability of heterologously expressed *Hco-pgp-2* to transport dyes in the presence of IVM and MOX differed. While IVM inhibited the transport of the utilized dyes in a concentration-dependent manner, dye efflux was not saturable with high concentrations of MOX [61]. In addition, the selection of *C. elegans* with either IVM or MOX revealed constitutive higher expression of *Cel-pgp-8* in the MOX-selected strain, while *Cel-pgp-10* appeared to be IVM specific [29].

Although members of the *H. contortus cyp* and ABC families have been described [62, 65], the fast development of next-generation sequencing technologies has enabled the generation of highly contiguous genomes. These techniques have facilitated the generation of an optimized, highly contiguous 283.4-Mbp chromosome-scale genome assembly of the *H. contortus* inbred-susceptible-Edinburgh (ISE) isolate [66]. 

The aim of this study is to re-characterize the *H. contortus cyp* family and compare these results with those of the described putative *cyp* genes [65]. In addition, the differential gene expression of the putative *cyp* and ABC transporters, which have been recently described in detail based on the current genome assembly reported by Mate et al. [62], was investigated using transcriptome data. For this purpose, fourth-stage larvae (L4) (of a highly ML-resistant *H. contortus* isolate were time-dependently exposed to IVM and MOX. Then, RNA sequencing was performed, and the reads were mapped to the recently published reference genome of MHco3(ISE).N1 *H. contortus* [66]. Finally, a comparative analysis of gene expression of *cyp* genes and ABC transporters in L4 was conducted as follows: (1) between the susceptible McMaster and the highly IVM- and MOX-resistant Berlin-selected isolate (BSI); (2) through a time course expression analysis for the BSI isolate during culture in the vehicle controls; and (3) differential gene expression analysis comparing the effects of IVM and MOX exposure on the resistant BSI isolate. The effects of IVM and MOX were not examined in the susceptible isolate since they were expected to predominantly result in detectable stress responses, as previously described for *C. elegans* [31].

## Methods

### Identification of *cyp* sequences and phylogenetic analysis

To identify putative *H. contortus* (PRJEB506) [66], *P. univalens* (PRJNA386823) [67], and *Ascaris suum* (PRJNA386823) [67] *cyp* genes, polypeptide sequences for all *C. elegans cyp* genes available in Wormbase were used in a translated Basic Local Alignment Search Tool nucleotide database (tBLASTn) search against the draft genome assemblies. The search was repeated with polypeptide sequences of the human CYP3A4 (P08684), CYP3A5 (P20815), CYP2E1 (P05181), CYP2D6 (P10635), CYP2C19 (P33261), CYP2C9 (P11712), CYP2C8 (P10632), CYP2B6 (P20813), CYP2A6 (P11509) and CYP1A2 (P05177) sequences, which are known to be primarily responsible for human drug metabolism [16]. The human peptide sequences were downloaded from the UniProt database. In the case of *H. contortus*, putative *cyp* sequences identified by Laing et al. [65] were additionally used in a tBLASTn search against the *H. contortus* draft genome (PRJEB506) [66] to assign the already characterized *cyp* genes to the updated genome and identify potential pseudogenes. All identified coding nucleotide sequences were translated with Expasy Translate [68] to control the open reading frame and compared to the according amino acid sequences. The peptide sequences were used to predict protein parameters [69] and the position of the conserved CYP cysteine heme iron ligand motif (accession number PS00086) [70].

### Phylogenetic analysis

The CYP protein sequences were aligned using the M-Coffee mode of T-Coffee [71] on the T-Coffee server [72] using default settings. A maximum-likelihood phylogenetic tree was constructed using IQ-TREE 1.6.12 [73] on the IQ-TREE server [74]. ModelFinder [75] was used to identify the best substitution model based on the lowest Bayesian information criterium value, and models with FreeRate heterogeneity were included in the model search. Branch support values were calculated using 1000 ultrafast bootstrapping pseudorepeats [76] and the Shimodaira-Hasegawa (SH) transformation of the likelihood ratio test [77]. The resulting phylogenetic tree was visualized using FigTree 1.4.4. No outgroup was used to root the tree.

### Parasites

This study included two isolates of *H. contortus* with differing susceptibilities to BZs and MLs.

1. *Haemonchus contortus* McMaster (*H.c.* McM), which was susceptible to IVM and MOX [78].
2. *Haemonchus contortus* BSI (*H.c.* BSI), which was highly resistant to thiabendazole, MOX, and IVM. This isolate was obtained from two sheep bought by the Institute for Parasitology and Tropical Veterinary Medicine from a farmer in Brandenburg (Havelland district), Germany, in 2018. A lamb received a treatment with MOX but parasites were not completely cleared, and eggs (< 10 eggs per gram) remained even after a second treatment. Fecal cultures were set up and L3 collected from these post-treatment samples and used to infect another lamb that was treated with MOX. This treatment had no effect on egg counts. This lamb was euthanized, and gravid female worms were carefully ground and mixed with feces from nematode-negative animals, and stored frozen for several days to destroy any nematode eggs that may still have been present. Larvae obtained from these larval cultures were used to establish the isolate by infecting two naïve lambs. The parasites have been propagated in lambs at least once per year since the isolation of *H.c.* BSI. According to the pyrosequencing assay used, the isolate has a frequency of > 80% of the F200Y polymorphism in the isotype 1 β-tubulin gene.

All isolates have been maintained at the Institute for Parasitology and Tropical Veterinary Medicine of the Freie Universität in Berlin for several years. All animal experiments were in agreement with the European directive 2010/63/EU and the German law (Tierschutzgesetz) and were approved by the responsible local authorities (LAGeSo Berlin) under the reference number H0337/17. Individual sheep were infected with approximately 6000 L3 of one isolate, and feces were collected for strongyle egg purification.

### Chemicals

Stock solutions of 10 mM IVM (I8898; Sigma-Aldrich, Germany) and MOX (33746; Sigma-Aldrich) were dissolved in 100% dimethyl sulfoxide (DMSO).

### Strongyle egg purification and larval development assay

Strongyle eggs were purified from fresh feces using a sucrose step gradient. Briefly, feces were homogenized and passed through a 100-μm sieve. Eggs in the flow-through were collected on a 25-μm sieve followed by centrifugation and flotation with saturated sodium chloride solution. Then, the egg suspension was placed on top of a sucrose step gradient containing 10%, 25%, and 40% of a saturated sucrose solution and centrifuged at 2000 *g* and 4 °C for 5 min. Eggs floated between the 10% and 25% layers. They were collected and washed with tap water.

The larval development assays were performed following the method described by Demeler et al. [79], with slight modifications regarding the addition of IVM and MOX. Stock solutions were serially diluted with DMSO. In the assay wells, a serial dilution leading to final concentrations of 0.05, 0.01, 0.05, 0.1, 0.5, 1, 5, and 10 µM of IVM and MOX, respectively, was used in assays with the isolate *H.c.* BSI. For the susceptible *H.c.* McM isolate, final concentrations of 0.001, 0.005, 0.01, 0.05, 0.1, 0.5, 1, 5 nM IVM and MOX, respectively, were used. Positive controls contained 50 µM MOX and IVM, respectively. Finally, the assays were performed with 30 µL (+ 200 µL deionized water) of IVM and MOX drug dilutions in each well to achieve the final concentrations. As a food source for larval development and inhibition of fungal growth, 50 µL of growth medium was added. The growth medium was prepared as a mixture containing 20 µL yeast/Earle's solution [9:1 mixture of 10 $\times$ concentrated Earle's solution (E7510; Sigma) and yeast extract, prepared by suspending 1 g yeast in 90 mL 0.9% NaCl and autoclaving for 20 min], 20 µL of 0.5 mg/mL amphotericin (A2942; Sigma) and 10 µL of 1.5 mg/mL lyophilized *Escherichia coli* K12 (EC1; Sigma). Finally, 20 µL of egg suspension, adjusted to contain 100–120 strongyle eggs, was added. The plates were sealed with parafilm to prevent evaporation and placed in an incubator for 7 days at 24 °C. After incubation, the assays were terminated by adding two drops of Lugol's iodine (100567; Merck, Germany) to each well. The eggs, L1, L2, and L3, were counted separately in each well, and the percentage of L3 was calculated. Concentration–response curves were calculated by fitting four-parameter logistic regression curves in GraphPad Prism 5.02. The top and bottom parameters were constrained to values between 0 and 100%. A comparison of half maximal effective concentration (EC_50_) values between isolates was performed using the sum of squares* F*-test.

### In vitro cultivation of L3 to L4

The in vitro protocol for cultivating *H. contortus* was performed by following the method described by Yilmaz et al. [14]. Briefly, L3 underwent exsheathment by treatment with sodium hypochlorite and were then washed with 0.9% NaCl solution by using a bottle-top sterile filter. Then, the larvae were transferred to a Baermann apparatus filled with pre-warmed 0.9% NaCl solution, and allowed to migrate through the mesh. The L3 were then collected in a 50-mL centrifugation tube, placed on a bottle-top filter, washed with axenization fluid at 37 °C and centrifuged at 90 r.p.m. for 3 h. Finally, the L3 were concentrated by filtration and transferred into cell culture flasks with supplemented RPMI 1640 culture medium at a density of approximately 2000 larvae mL^−2^ and incubated in 20% CO_2_ at 37 °C for 3 days. On day 3, the medium was replaced by fresh culture medium and incubated for 2 more days. The development to L4 was microscopically tracked (Olympus CK2 inverse microscope; Olympus, Japan).

### IVM and MOX exposure of *Haemonchus contortus* L4

Larvae were microscopically tracked (Olympus CK2 inverted microscope) to ensure that at least 80% had molted to the L4 stage and displayed active pharyngeal pumping. Then, L4 of a single batch were split into nine groups of approximately 100,000 L4, and each group placed in a T75 cell culture flask. Three groups were exposed to IVM (100 nM) for 3 h, 6 h, or 12 h, three other groups to MOX (100 nM) for 3 h, 6 h, or 12 h, and the remaining three groups served as the vehicle control and were incubated with 0.05% DMSO for 3 h, 6 h, or 12 h. After incubation, L4 were washed with 4 °C ice-cold 0.9% NaCl solution via rapid filtration on a 200-µM bottle-top filter and collected from the top of the filtration membrane into a 50-mL centrifugation tube. Larvae were pelleted by centrifugation at 3214 *g* 4 °C for 5 min, resuspended in cold 0.9% NaCl solution, and again pelleted. The supernatant was discarded, the pellet transferred to a NucleoSpin® Bead Tube Type A (Macherey Nagel) lysis tube, and 900 µL RA1 lysis buffer (NucleoSpin® RNA kit from Macherey Nagel) was added. The larvae were then homogenized using a SpeedMill P12 (Analytik Jena) for three cycles of 1-min duration. Then, 9 µL β-mercaptoethanol was added to each of the samples, which were vortexed and then frozen at − 80 °C until RNA extraction.

### RNA extraction and RNA sequencing

Total RNA was extracted using the NucleoSpin® RNA XS kit (Macherey Nagel) according to the manufacturer's instructions. The quantity of RNA was measured for each sample in a Qubit Fluorometer (Invitrogen) using the Qubit™ RNA HS Assay Kit (Thermo Fisher).

RNA library preparation and sequencing were performed by Novogene. The RNA sequencing library was sequenced on the Illumina platformNovaseq, and 150-base pair paired-end reads were generated.

### Transcriptome data processing

For one of the samples of BSI isolate incubated with DMSO for 12 h, a forward read fastq file was corrupted during download; this was noticed only after the data had been deleted from the server of the sequencing facility, thus only five replicates were included in the analysis. All raw data were deposited in the Sequence Read Archive (previously known as the Short Read Archive) under Bioproject accession number PRJNA1105765. Raw reads were adapter and quality trimmed with Cutadapt (version 2.10) [80]. Trimmed reads were subsequently mapped to the reference genome of *H. contortus* (PRJEB506.WBPS15) [66] with STAR (version 2.7.5c) [81] and then aggregated to gene counts with the tool featureCounts (version 2.0.1) [81]. The exact command lines for each step are listed in additional file S1 Table S11.

### Differential gene expression analysis

Differential gene expression analysis was performed using egdeR [82] and DESeq2 [83] R package (R version 4.2.0: RStudio-2022.02.3+492) to normalize raw counts by the trimmed mean of* M*-values method and by the geometric mean for each gene across all samples (DESeq2). Both normalization approaches revealed suitable methods to demonstrate transcriptomic alterations with. However, DESeq2 was chosen for visualization to display the greater count variance between genes within the CYP and ABC transporter families. In the following, mean normalized counts were statistically analyzed and visualized with GraphPad Prism 5.0.3. Statistical analysis was carried out to compare the gene expression level of target genes between treatment groups within one isolate and between the resistant BSI and susceptible McMaster isolates without drug exposure by using two-way ANOVA with the Bonferroni post hoc test.

## Results

### Effects of IVM and MOX on *Haemonchus contortus* larval development

**Fig. 1:**
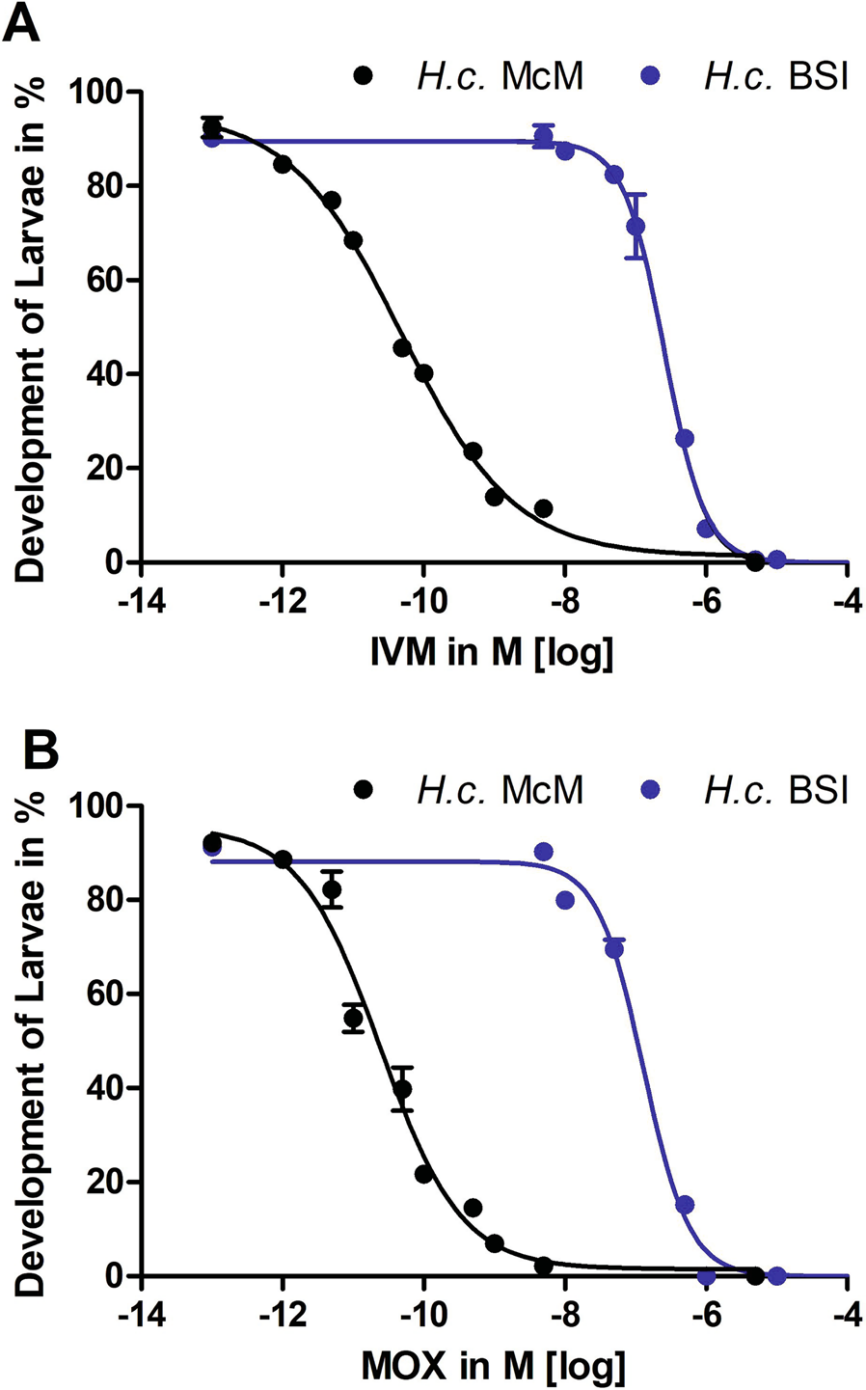
Concentration–response curves of the larval development assay for the *Haemonchus contortus* Berlin-selected isolate (*H.c. BSI*) against ivermectin (*IVM*) (**A**) and moxidectin (*MOX*) (**B**). The drug-susceptible McMaster isolate (*H.c. McM*) was included for comparison.* Error bars* show the SEM of at least three replicates. For both IVM and MOX, the half maximal effective concentration (*EC*_50_) values for* H.c. McM* and* H.c. BSI* were significantly different from each other (*P* < 0.0001)

Phenotypic resistance of the *H.c.* BSI against IVM and MOX was determined using larval development assays (Fig. 1) before calculating EC_50_ values (Table 1) using four-parameter logistic regressions. The coefficient of determination (*R*^2^) ranged from 0.95–0.99 for all drugs and isolates, indicating a good fit for all assays performed. The BSI isolate showed an EC_50_ value for IVM of 258 nM, which was almost double that for MOX (130 nM) (Table 1). For both drugs, the EC_50_ values were significantly higher than for the susceptible McMaster isolate, with very high resistance ratios (RRs) of more than 5000 (Table 1). The BSI isolate showed almost identical shifts in EC_50_ values compared to the McMaster for both MLs.

**Table 1:** The half maximal effective concentration (*EC*_50_) values with 95% confidence intervals (*CI*) and the resistance ratios (*RR*) for ivermectin (*IVM*) and moxidectin (*MOX*) for the susceptible (*H.c. McMaster*) and resistant (*H.c.* Berlin-selected isolate; *H.c. BSI*) isolates of *Haemonchus contortus*

Isolate	Drug	EC_50_ (nM)	95% CI	*R*^2^	*RR*^a^
*H.c.* McMaster	IVM	0.0491	0.0394–0.0613	0.9888	
	MOX	0.0243	0.0179–0.0329	0.9585	
*H.c*. BSI	IVM	258.2	214.3–310.9	0.9504	5258
	MOX	130	111.2–151.9	0.9839	5349

*R*^2^ Coefficient of determination

^a^*RR *for the comparison with the McMaster isolate for the same drug

### Comparison of the cytochrome P450 family of *Haemonchus contortus* with that of other nematodes

Due to the availability of a higher quality *H. contortus* reference genome, reported by Doyle et al. [66], compared to prior genome assemblies used for *cyp* identification reported by Laing et al. [65], we decided to reanalyze the *H. contortus* cytochrome P450 repertoire.

**Fig. 2:**
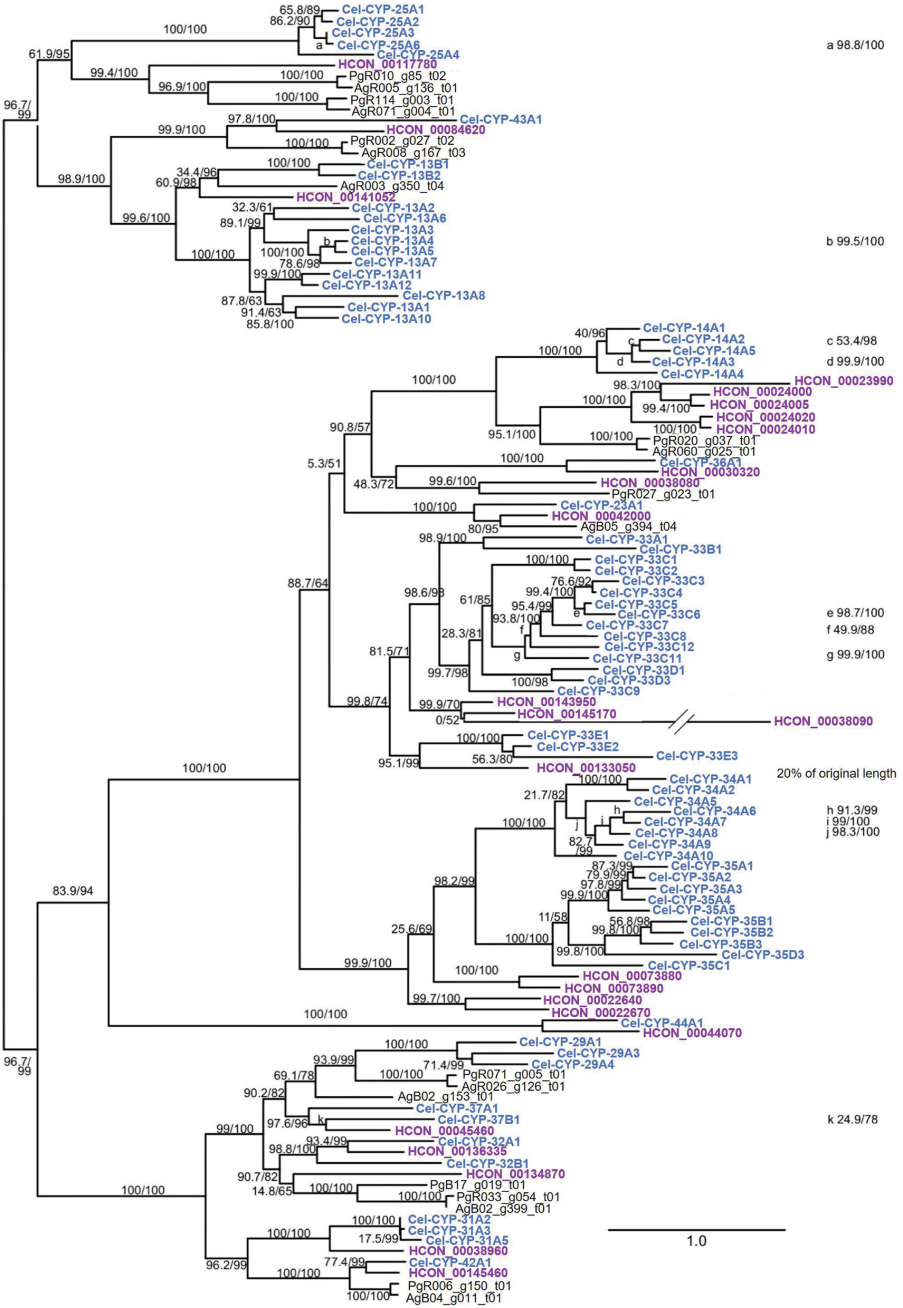
Phylogenetic analysis of cytochrome P450 (*CYP*) protein sequences from *Haemonchus contortus* (*HCON*; violet), *Caenorhabditis elegans* (*Cel*; dark blue), *Ascaris suum* (*Ag*) and *Parascaris univalens* (*Pg*). An unrooted maximum-likelihood phylogenetic tree was constructed in IQ-TREE. Node support values represent the Shimodaira-Hasegawa (*SH*) transformation results of the likelihood ratio test (before the slash) and ultrafast bootstrapping (after the slash). The scale bar represents 1.0 substitutions per site. Accession numbers were derived from the following genome assemblies: *PRJEB506*, for *H. contortus*; *PRJNA62057*, for *A. suum*; *PRJNA386823*, for* P. univalens*

A total of 25 *H. contortus* CYP proteins were identified based on BLAST protein (BLASTp) searches against 84 *C. elegans*, 10 *A. suum* (Additional file S1: Table S1), and nine *P. univalens* (Additional file S1: Table S2) orthologous proteins. The identified sequences were analyzed by determining the CYP heme iron ligand motifs (Additional file S1: Table S3) to screen for sequence artifacts with similarity to CYPs but missing this crucial motif. The obtained sequences (Additional file S1: Table S4) were assigned to the CYP families based on a phylogenetic analysis that included the BLASTp matches of the four nematode species (Fig. 2).

As expected, as a consequence of the high number of CYP proteins encoded in the *C. elegans* genome, and in accordance with Laing et al. [65], multiple *cyp* gene families were expanded in *C. elegans*, but have only a single member in *H. contortus*. The *cyp* gene family is much smaller in species of the genus* Ascaris* than in *H. contortus*. In accordance with the phylogenetic analysis, the 25 *H. contortus cyp* genes were assigned to 13 families (Fig. 2). The majority of these families also included members of *C. elegans*. However, two *H. contortus* CYP families were placed in a group with two *C. elegans* CYP families (CYP34 and CYP35) without any one-to-one orthology relationship between the four groups. No member of the *C. elegans* CYP family (CYP29) was found in *H. contortus*. This might have been partly due to missing members, since not all *cyp* genes of the closely related species *A. suum* and *P. univalens* were found in closely related pairs. In Fig. 2., seven of these pairs can be identified, while three *A. suum* and two *P. univalens cyp* genes occurred without a clear ortholog for the other species, suggesting that the latter had been missed due to data missing from the genome assembly.

The different CYP families can be divided into one group with only one member from the different nematode species and a second group comprising multiple members from the different species. The CYP families with only one member per species include CYP23, CYP42, CYP43. The CYP36 and CYP44 families were also found to comprise single-copy genes, but only for *C. elegans* and *H. contortus*, while these were not found in the ascarids. HCON00038080 from *H. contortus* and its ortholog PgR027_g023_t01 in *P. univalens* represent a pair of orthologs for which no family members were found in *C. elegans* and *A. suum*.

Some CYP families showed strong diversification in *C. elegans*, while only one member was found in the parasitic nematodes. The CYP13 family has 13 members in *C. elegans*, divided into two subfamilies. While there are no orthologs of the 11 CYP13A subfamily members, HCON_00141052 and AgR003_g350_t04 cluster with the two CYP13B subfamily members. The BLAST searches did not detect an ortholog in the *P. univalens* genome assembly.

Diversification of the CYP32 family was observed in all three nematode groups, with two members in *C. elegans*, *H. contortus*, and *P. univalens*, while only a single sequence was found in the *A. suum* genome. The CYP14 family showed diversification in *C. elegans* and *H. contortus* but not in the ascarids. CYP33 family members showed strong diversification in *C. elegans* (18 members divided into five subfamilies), while a total of four members in two subfamilies were found in *H. contortus*. One *H. contortus* subfamily with three members was found in a sister position to *C. elegans* CYP subfamilies CYP33A-CYP33D. In contrast, a single *H. contortus* CYP HCON_001330050 occurred in a sister position to the CYP33E subfamily of *C. elegans*. There was no evidence that the CYP33 family might occur in ascarid nematodes. The subfamilies CYP34 (eight members in *C. elegans*) and CYP35 (10 members in *C. elegans*) were found in a group together with two *H. contortus* subfamilies, each with two members, i.e., HCON_00073880 and HCON_0073890 in the former and HCON_00022640 and HCON_00022670 in the latter subfamily. No closely related CYP sequences were found in the ascarids. Similarly, although it had a much lower level of diversification, the CYP31 family (three members in *C. elegans*) and CYP37 family (two members in *C. elegans*) had only one member in *H. contortus* but were absent from the ascarid genome assemblies. The CYP25 gene family is quite diverse in *C. elegans*, with five members, while *H. contortus* contains only a single member of this gene family. In contrast, two family members were found in both ascarid genomes. The CYP29 family has three members in *C. elegans* but only individual orthologs in both ascarids, while an ortholog from *H. contortus* was not observed.

### RNA sequencing analysis

RNA sequencing was performed with the IVM- and MOX-resistant *H. contortus* BSI and the drug-susceptible *H. contortus* McMaster isolate to identify constitutive as well as IVM- and MOX-inducible expression of CYPs and ABC transporters.

Preliminary incubation experiments were conducted at four different IVM and MOX concentrations (1, 10, 50, and 100 nM) to determine the drug dose giving the maximum induction of expression of resistance-associated genes in *H. contortus* BSI. To achieve this, the L4 should still exhibit phenotypic pharyngeal activity and motility, i.e. they should not be paralyzed. The microscopic analysis demonstrated decreased phenotypic parameters (thrashing and rolling) with increased drug concentrations, and for 12 h (Additional file S1. Fig. S1 for IVM; Fig. S2 for MOX). Since only a minor treatment effect was observed and the larvae were not paralyzed, the induction experiments for RNA sequencing were conducted with 100 nM IVM and 100 nM MOX. No incubation experiments were conducted with *H. contortus* McM as this ML-susceptible isolate was expected to become paralyzed with the tested concentrations.

Between 88 and 91% of *H. contortus* McMaster and *H. contortus* BSI reads mapped to the reference genome PRJEB506 [66] (Additional file S1: Fig. S3). Transcriptomic differentiation between treatment groups, incubation times, and replicates was assessed using principal component analysis (Additional file S1: Fig. S4). Analysis by treatment group and incubation time revealed less defined clustering and greater variance among all samples. In contrast, samples clustered well by replicate, which corresponded to the sample processing batch. Therefore, this batch effect was considered a random factor in the DESeq2 analysis.

### Differences of cytochrome P450 gene expression between an IVM- and MOX-resistant and IVM- and MOX-susceptible *Haemonchus contortus* isolate

To investigate which CYP enzymes were constitutively highly expressed in the resistant *H. contortus* BSI isolate compared to the susceptible *H. contortus* McMaster, a comparative analysis of the mean normalized counts determined by DESeq 2 was performed. Hence, vehicle controls incubated with 0.05% DMSO for 3 h for both isolates were selected for the following analysis.

**Fig. 3:**
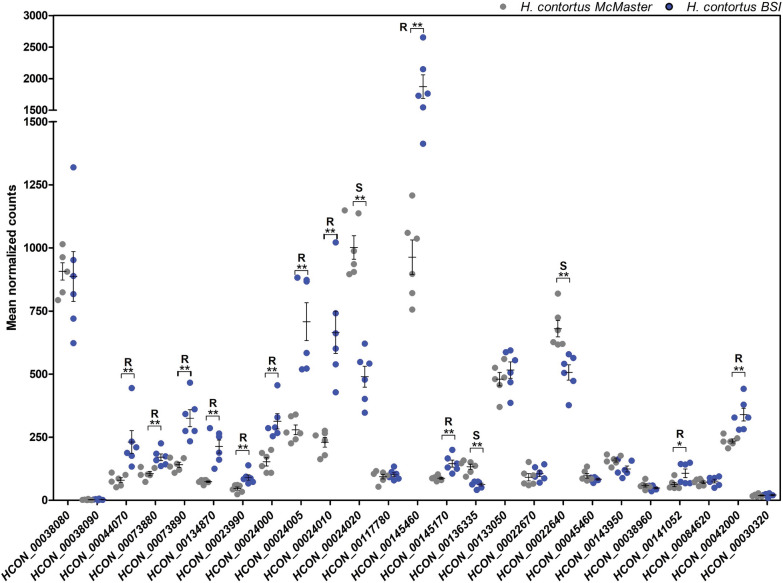
Relative expression (mean normalized counts) of cytochrome P450 genes identified by RNA sequencing. The data were obtained from six biological replicates per isolate exposed to 0.05% dimethyl sulfoxide (DMSO) for 3 h. *S* Expression was higher in the susceptible isolate,* R* expression was higher in the resistant isolate. Gene model identifiers (IDs) correspond to the *Haemonchus contortus* genome assembly PRJEB506 [66].* Blue* *H. contortus* BSI,* grey* *H. contortus* McMaster. Statistically significant higher gene expression for *H. contortus* BSI (*blue*) is indicated by R, and that for *H. contortus* McMaster by an S. *P*-values were determined by Mann–Whitney test (* *P*-value < 0.05, ** *P*-value < 0.01)

L4 of the resistant *H. contortus* BSI isolate showed higher constitutive expression of 12 *cyp* genes (Fig. 3, Additional file S1: Table S5), including HCON_0044070 (log_2_FC 1.41, *P* < 0.01), HCON_0073880 (log_2_FC 0.66, *P* < 0.01), HCON_0073890 (log_2_FC 1.17, *P* < 0.01), HCON_00134870 (log_2_FC 1.46, *P* < 0.01), HCON_0023990 (log_2_FC 0.92, *P* < 0.01), HCON_0024000 (log_2_FC 1.01, *P* < 0.01), HCON_0024005 (log_2_FC 1.30, *P* < 0.01), HCON_0024010 (log_2_FC 1.48, *P* < 0.01), HCON_00145460 (log_2_FC 0.93, *P* < 0.01), HCON_00145170 (log_2_FC 0.70, *P* < 0.01), HCON_0042000 (log_2_FC 0.51, *P* < 0.01), and HCON_00141052 (log_2_FC 0.68, *P* < 0.05).

In contrast, HCON_00024020 (log_2_FC − 1.06, *P* < 0.01), HCON_00136335 (log_2_FC − 1.13, *P* < 0.01), and HCON_0022640 (log_2_FC − 0.44, *P* < 0.01) showed significantly lower expression levels in the resistant *H. contortus* BSI isolate compared to the susceptible *H. contortus* McMaster.

### Time course of cytochrome P450 enzyme expression in the *Haemonchus contortus*-resistant BSI isolate in the absence of IVM and MOX

Among the *cyp* genes, HCON_00145460, HCON_0038080, HCON_0024010, and HCON_0024005 displayed the highest transcript levels at 3, 6, and 12 h in the control group, ranging from a mean normalized count of 680 for HCON_0024010 at 3h to 1940 for HCON_00145460 at 3 h (Additional file S1: Fig. S5). Transcript levels for HCON_00145460 and HCON_0038080 slightly decreased from 3 to 12 h, but these changes were not statistically significant.

A comparison of the basal transcript levels revealed no significant differences over time in any of the *cyp* genes (Additional file S1: Table S6 and Fig. S6).

### Inducible gene expression of cytochrome P450 enzymes in the *Haemonchus contortus* BSI following incubation with IVM and MOX for 3, 6, and 12 h

To elucidate the inducibility of cytochrome P450 gene expression in response to IVM and MOX and to differentiate between early and late treatment effects, L4 of the IVM- and MOX-resistant *H. contortus* BSI isolate were incubated with the MLs for 3, 6 and 12 h.

**Fig. 4:**
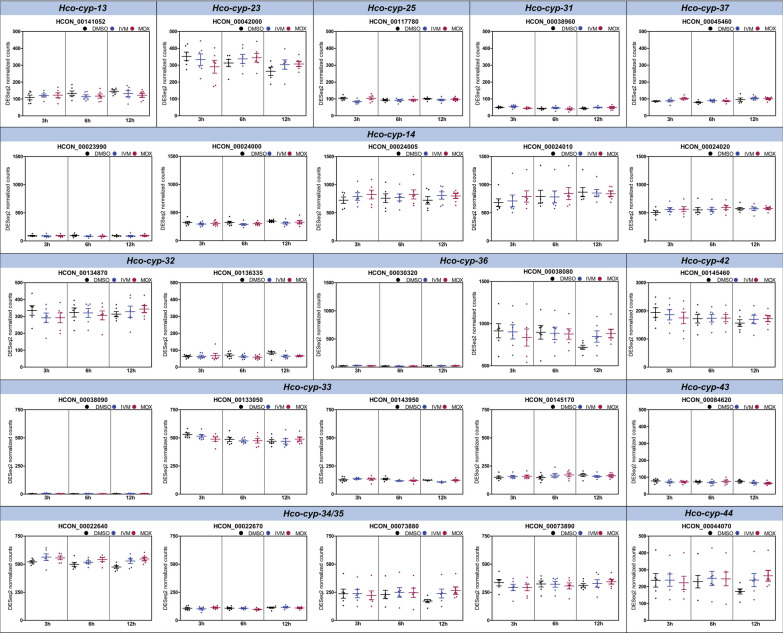
Time course of differential gene expression of *Haemonchus contortus* cytochrome P450 enzymes based on RNA sequencing. Scatter plot showing DESeq2 normalized counts of 100 nM IVM, 100 nM MOX, and 0.05% DMSO-treated *Haemonchus contortus* BSI fourth-stage larvae (L4) at 3, 6, and 12 h.* Horizontal lines* represent means,* error bars* the SD. Data were obtained from six biological replicates. Statistical analysis to compare the gene expression level of target genes between treatment groups (factor 1) and over time (factor 2) was carried out using a two-way ANOVA followed by a Bonferroni post hoc test. No significant upregulation of any *cyp* was identified following the incubation of larvae with IVM and MOX vs DMSO. Gene model IDs correspond to the *H. contortus* genome assembly PRJEB506 [66].* Black* DMSO control,* Blue* IVM,* Pink* MOX

IVM and MOX incubation of *H. contortus* BSI did not significantly change expression level of any of the *cyp* genes compared to the DMSO vehicle control in terms of mean normalized counts (Fig. 4, Additional file 1: Table S7). No significant differences in expression profiles between the treatment groups were identified either.

### Differences in ABC transporter gene expression between an IVM- and MOX-resistant and a susceptible *Haemonchus contortus* isolate

Transcripts of 50 *H. contortus* ABC transporter genes have been described in detail by Mate et al. [62] based on the most recent reference *H. contortus* reference genome (PRJEB506.WBPS15). The constitutive expression of these ABC transporters in the resistant *H. contortus* BSI isolate was compared to that of the susceptible *H. contortus* McMaster by conducting a comparative analysis of the mean normalized counts determined by DESeq 2. Vehicle controls from both isolates were incubated with 0.05% DMSO for 3 h for the analysis.

**Fig. 5:**
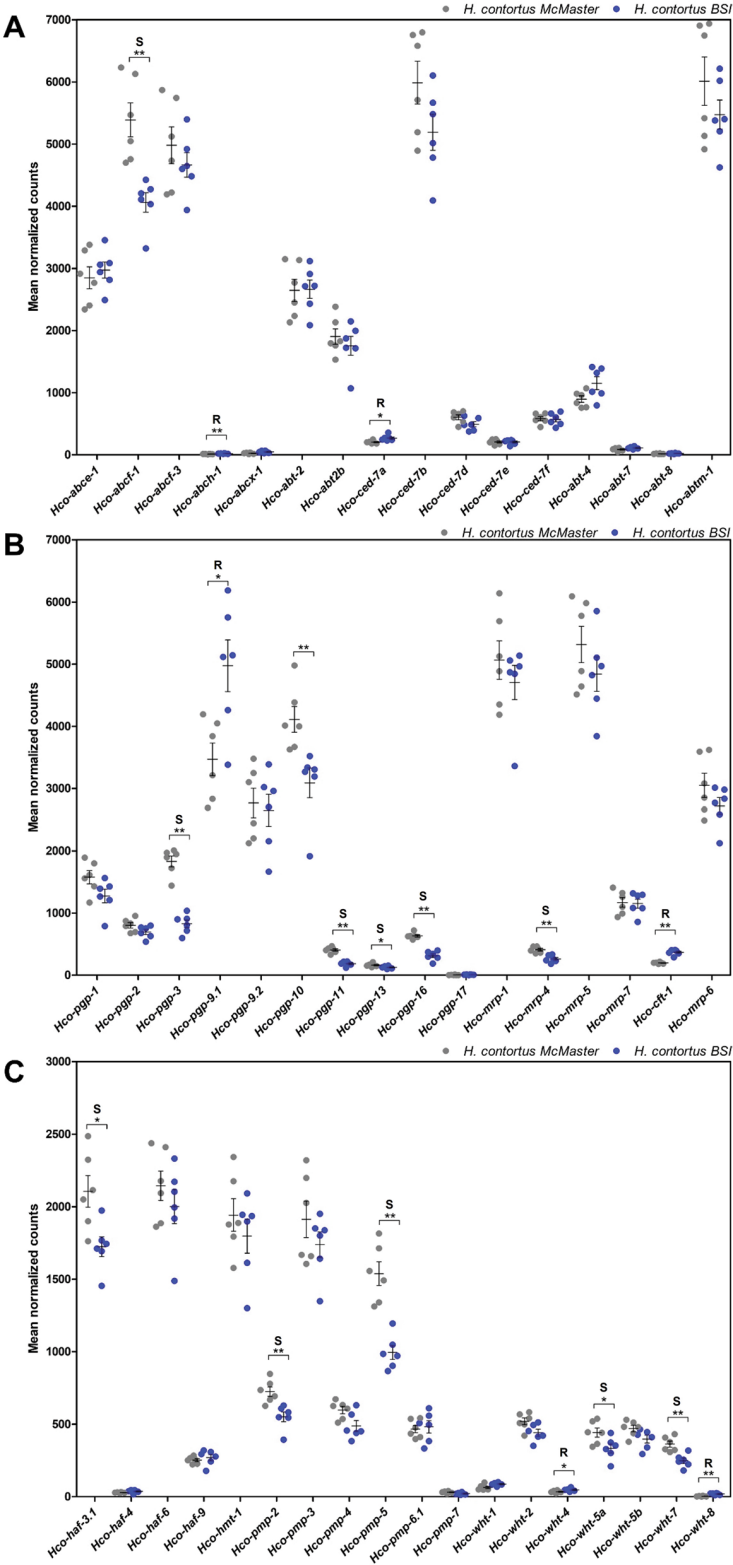
Relative expression (mean normalized counts) of **A** *abc* and *abt*, **B** *pgp* and *mrp*, **C** *haf*, *hmt*, *pmp* and *wht* transcripts in *Haemonchus contortus* BSI (*blue*) and *H. contortus* McMaster (*grey*) L4. The data were obtained from six biological replicates per isolate exposed to 0.05% DMSO for 3 h.* Horizontal lines* represent means and *error bars* the SD. *P*-values were determined by Mann–Whitney (* *P*-value < 0.05, ** *P*-value < 0.01). * S* Expression was higher in the susceptible isolate, * R* expression was higher in the resistant isolate. Statistically significant higher gene expression is indicated by an *R* for *H. contortus* BSI and an* S* for *H. contortus* McMaster. ABC transporter class [including ATP-binding cassette (ABC) transporter class E–H, and ABC transporter extended]; *abt* ABC transporter family, *abtm* ABC transporter mitochondrial, *haf* half transporter, *hmt* heavy metal tolerance factor, *pmp* peroxisomal membrane protein-related, *wht* white *Drosophila*-related ABC transporter

Notably, transcripts of six ABC transporter genes were significantly highly expressed in the resistant BSI compared to the susceptible McMaster isolate (Fig. 5, Additional file S1: Table S8), including *Hco-abch-1* (log_2_FC 0.71, *P*-value < 0.01), *Hco-ced-7a* (log_2_FC 0.38, *P*-value < 0.05), *Hco-wht-4* (log_2_FC 0.43, *P*-value < 0.05), *Hco-wht-8* (log_2_FC 2.37, *P*-value < 0.01), *Hco-cft-1* (log_2_FC 0.89, *P*-value < 0.01), and *Hco-pgp-9.1* (log_2_FC 0.49, *P*-value < 0.05).

In addition, the comparative analysis demonstrated that 12 genes were significantly lower expressed in the resistant BSI isolate. These represented *Hco-abcf-1* (log_2_FC − 0.41, *P*-value < 0.01), *Hco-haf-3.1* (log_2_FC − 0.29, *P*-value < 0.05), *Hco-pmp-2* (log_2_FC − 0.41, *P*-value < 0.01), *Hco-pmp-5* (log_2_FC − 0.64, *P*-value < 0.01), *Hco-wht-5a* (log_2_FC − 0.44, *P*-value < 0.05), *Hco-wht-7* (log_2_FC − 0.57, *P*-value < 0.01), *Hco-pgp-3* (log_2_FC − 1.17, *P*-value < 0.01), *Hco-pgp-10* (log_2_FC − 0.44, *P*-value < 0.01), *Hco-pgp-11* (log_2_FC − 1.17, *P*-value < 0.01), *Hco-pgp-13* (log_2_FC − 0.38, *P*-value < 0.05), *Hco-pgp-16* (log_2_FC − 1.05, *P*-value < 0.01), and *Hco-mrp-4* (log_2_FC − 0.68, *P*-value < 0.01) (Fig. 5, Additional file 1: Table S8).

### Time course of ABC transporter gene expression in the *Haemonchus contortus* resistant BSI isolate

Comparison of basal transcript levels based on mean normalized counts from RNA sequencing revealed no significant differences for ABC transporter in the *H. contortus* BSI resistant isolate control group over 3, 6, and 12 h (Additional file S1: Table S9 & Fig. S7 & Fig. S8).

### Inducible ABC transporter gene expression in the *Haemonchus contortus* BSI following incubation with IVM and MOX for 3, 6, and 12 h

**Fig. 6:**
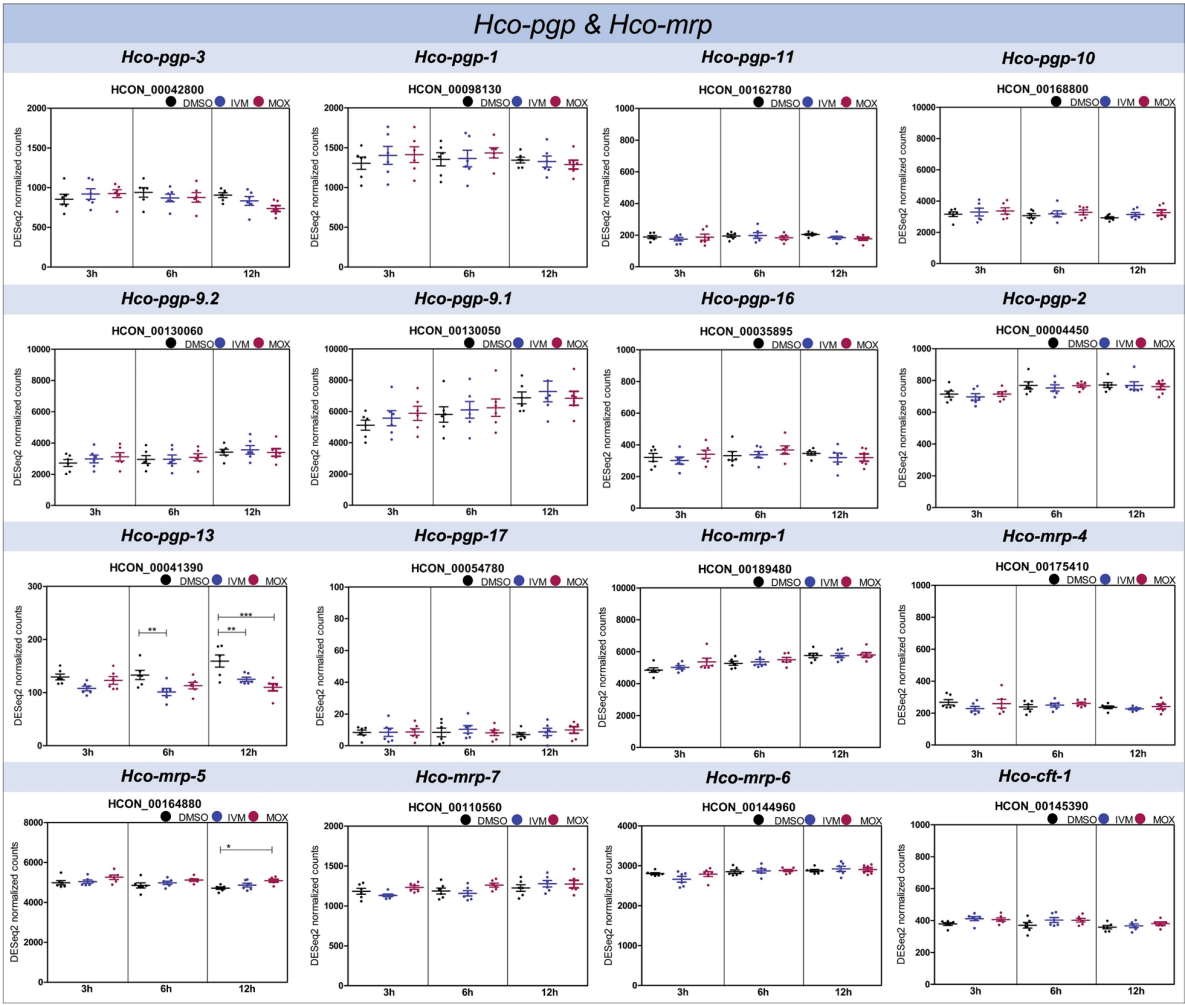
Time course of differential gene expression analysis of *Haemonchus contortus* P-glycoproteins (*pgp*) and multidrug resistance proteins (*mrp*) based on RNA sequencing. Scatter plot shows mean normalized counts (DESeq2) of 100 nM IVM, 100 nM MOX, and 0.05% DMSO-treated *Haemonchus contortus* BSI L4 for 3, 6, and 12 h. Data were obtained from six biological replicates.* Horizontal lines* represent means and* error bars* the SD. Statistical analysis to compare the gene expression level of target genes between treatment groups (factor 1) and over time (factor 2) was carried out using a two-way ANOVA followed by a Bonferroni post hoc test (* *P*-value < 0.05, ** *P*-value < 0.01, *** *P*-value < 0.001). *H. contortus* ABC transporter genes are grouped into gene families, in accordance with Mate et al. [62]. Gene model IDs correspond to the *H. contortus* genome assembly PRJEB506 [66].* Black* DMSO control,* blue* IVM,* pink* MOX

For ABC transporters, differential gene expression analysis depicted significant changes due to IVM and MOX treatment only for *Hco-pgp-13* and *Hco-mrp-5* (Fig. 6, Additional file S1: Fig. S9).

Transcript levels for *Hco-pgp-13* showed significant downregulation following incubation with IVM and MOX compared to DMSO. While incubation with IVM led to downregulation as early at 6 h, which remained stable at 12 h, a MOX-dependent effect was first determined at 12 h. However, no significant differences in transcript levels of *Hco-pgp-13* were identified between the IVM and MOX treatment groups. In contrast, *Hco-mrp-5* was significantly upregulated upon 12 h of MOX exposure (log_2_FC 0.03, *P*-value < 0.05), while exposure to IVM did not result in any significant up- or downregulation.

Although *Hco-pgp-9.1* was constitutively highly expressed in the resistant BSI isolate compared to the susceptible McMaster isolate at 3 h [5], and had the highest basal transcript level among the ABC transporters at 3, 6, and 12 h (Additional file S1: Fig. S7), no inducible expression pattern in response to IVM and MOX was observed. Whether constitutively higher expression in the BSI isolate persisted up to 12 h remains unclear.

## Discussion

An increasing number of studies suggest that ML resistance of *H. contortus* [10, 15, 62, 64] and other nematode species, such as *C. elegans* [29], *C. oncophora* [57, 84], *T. circumcincta* [33] and *P. univalens* [58, 85], are linked to pharmacokinetic-related defense mechanisms, such as biotransformation enzymes, including CYPs, and efflux transporters such as PGPs. Moreover, data from natural populations [86–88], artificial drug selection [41, 89], and transgenic expression of single candidate genes [36, 58, 59] indicate that IVM and MOX resistance exhibit slight mechanistic differences related to their pharmacological properties. While resistance against IVM is proposed to develop faster than that against MOX [40], MOX efficacy is maintained at higher levels when ML resistance develops [29, 90, 91].

Although several studies have been conducted to investigate the basal transcript levels of CYP and ABC transporter-encoding genes in *H. contortus*, a comprehensive picture of the whole *cyp* family, and a comparative analysis of the inducible expression of *cyp* genes and ABC transporters by IVM and MOX, are far from complete. Hence, the current work was focused on the CYP and ABC-transporter superfamilies and aimed to analyze the transcript levels in *H. contortus* isolates with different degrees of susceptibility to IVM and MOX. Secondly, inducible expression in response to IVM and MOX was investigated in a time-dependent manner.

For this purpose, the highly IVM- and MOX-resistant *H. contortus* BSI isolate was compared with the well-known drug-susceptible McMaster isolate. Since the BSI isolate has never been used in experimental studies before, its phenotypic resistance level against IVM and MOX was determined using the larval development test. The observed RRs, at above 5000, are extremely high. In a previous study using exactly the same setup for the larval development test on different *C. oncophora* isolates, RRs 6.5 and 9.8 higher than those of the susceptible reference isolate were found [92]. The exceptionally high RRs for IVM and MOX suggest that, in the isolate under discussion here, some fundamentally different resistance mechanisms might contribute to its phenotype, and that results might not be representative for field populations of *H. contortus* or closely related nematode species.

The comparative analysis of CYP transcripts in L4 of *H. contortus* revealed higher constitutive expression of 12 *cyp* genes (HCON_0044070, HCON_0073880, HCON_0073890, HCON_00134870, HCON_0023990, HCON_0024000, HCON_0024005, HCON_0024010, HCON_00145460, HCON_00145170, HCON_0042000, HCON_00141052) in the resistant BSI isolate compared to the susceptible McMaster isolate. Among these, HCON_0145460 demonstrated the highest basal expression in the resistant isolate over 12 h. However, the differences in expression levels between the isolates were rather small, in the range of log_2_FC 0.51–1.48 (corresponding to fold changes of 1.42–2.79). Moreover, no significant up- or downregulation of *cyp* genes following exposure to either ML for up to 12 h was observed.

The phylogenetic analysis performed here demonstrated that HCON_00145170 represents an ortholog of the *C. elegans cyp-33* subfamily that is known to be xenobiotically inducible [21]. Although no study has yet investigated the role of this particular *H. contortus cyp* in ML metabolism, or its potential contribution to resistance, its involvement in *C. elegans* detoxification mechanisms has been shown by Menzel et al. [21]. In addition, it has demonstrated that the *C. elegans cyp-33* subfamily play a role in lipid metabolism and fat mobilization [93].

Another *cyp* subfamily that potentially plays a role in ML resistance is *Hco-cyp-34/35*, which includes HCON_73880 and HCON_0073890. The orthologous *C. elegans cyp-35A* subfamily revealed xenobiotic inducibility similar to that of members of the *C. elegans cyp-33* subfamily. *Caenorhabditis elegans cyp-35A* and *cyp-35C* subfamily members showed concentration-dependent induction in response to atrazine, PCB52, fluoranthene, and lansoprazole [22]. A MOX-selected *C. elegans* strain was shown to exhibit a 4.8-fold higher constitutive expression of *Cel-cyp-35A1* relative to wild-type worms. Interestingly, this effect was only observed for the MOX-selected strain, not for the IVM-selected strain [29]. *Hco-cyp-13A11* has been shown to have specificity for some, but not all, MLs, as its transgenic expression in *C. elegans* decreased susceptibility to IVM (fourfold), IVM-aglycone (twofold), selamectin (threefold), and showed a slight effect for doramectin, but no effect for MOX and eprinomectin [36]. Transcript levels of *Hco-cyp-13A11* (HCON_00141052) were also slightly elevated for the resistant BSI isolate (1.60-fold compared to McMaster) in the current study, while differential gene expression upon ML exposure was not observed.

Significantly higher expression of *Cel-cyp-37B1* (5.7-fold) in the IVM-selected strain compared to wild-type worms was revealed by Menez et al. [29], while a similar effect was not detected after selection with MOX [29]. *Cel-cyp-37B1* was shown to be mainly expressed in the intestine, which is the prime site of detoxification in nematodes [92, 94]. However, constitutively higher expression of the *Cel-cyp-37B1* orthologue HCON_0045460 in the BSI resistant *H. contortus* was not found in the current work.

In contrast to *Cel-cyp-37B1*, elevated expression levels of *Cel-cyp-14A2* and *cyp-14A5*, in both IVM and MOX-selected strains, were reported by Menez et al. [29]. The *H. contortus* orthologs HCON_0023990, HCON_0024000, HCON_0024005, and HCON_0024010 were constitutively highly expressed in the BSI isolate in the present study. In contrast, none of the *cyp* genes demonstrated IVM inducibility in the resistant BSI isolate, unlike HCON_0024010, as shown in a study by Kellerova et al. [10], utilizing the susceptible *H. contortus* ISE isolate. Inducible overexpression of *Cel-cyp-14A5* was also confirmed by Mori et al. [95] after a single exposure to xenobiotics for 24 h.

However, with regard to other studies that investigated differential expression between isolates of different resistance status [31, 62, 96], it appears equally likely that constitutively higher expression of *H. contortus cyp* genes can also drive resistance. Constitutive overexpression has the potential to show protective effects at early stages upon exposure, while achieving considerable levels of functional enzymes via expression induction can take several hours. Considering a scenario in which multiple mechanisms contribute to the overall resistance level, higher constitutive transcript levels of multiple CYPs might well contribute to resistance without necessarily being a dominant factor.

Indeed, the stepwise selection of *C. elegans* with IVM and MOX resulted in constitutively higher expression of various *cyp* genes [29]. The results reported by Menez et al. [29] align with those of the present study, and support the hypothesis of constitutively higher expression of various *cyp* genes as a strategy in the evolution of ML resistance. Continuous but insufficiently high exposure of infected animals, such as sheep, to MLs, is often reported, and is considered a major reason for *H. contortus* ML-resistance selection [5, 97]. Thus, elevated expression profiles of *cyp* genes in resistant compared to susceptible isolates but not inducible *cyp* gene expression, may be the successful survival strategy that has evolved. In addition, the potential contribution of other drug-metabolizing enzymes that were shown to be constitutively highly expressed in IVM-selected *C. elegans* [29], such as glutathione *S*-transferases, needs to be considered.

Further investigation of the role of tissue-specific *cyp* expression and affinity towards IVM and MOX is needed, e.g., by ablation of multiple genes by utilizing loss-of-function strains to investigate if drug susceptibility is increased.

In the study performed here, not only were *cyp* genes shown to be constitutively highly expressed in the resistant BSI isolate, but the comparative analysis of ABC superfamily member transcripts in L4 also revealed elevated levels of *Hco-abch-1*, *Hco-ced-7a*, *Hco-wht-4*, *Hco-wht-8*, *Hco-cft-1*, and *Hco-pgp-9.1* in the resistant BSI isolate compared to the susceptible isolate. Moreover, *Hco-pgp-9.1* demonstrated the highest basal transcript level at 3, 6, and 12 h among all ABC transporter genes, although it did not reveal significant up- or downregulation following exposure to either ML for up to 12 h.

PGP-9 is a P-glycoprotein known to modulate IVM sensitivity in *H. contortus* [11, 62, 63, 96, 98, 99] and other nematode species [58, 85, 100]. IVM and abamectin demonstrated an inhibitory effect on the ability of transgenically expressed *Hco-pgp-9.1* to transport rhodamine 123 [63]. The *C. elongatus* Pgp-9 expressed in a *Saccharomyces cerevisiae* model was shown to protect against the antimycotic ketoconazole; this effect was counteracted in the presence of IVM and eprinomectin, but not in the presence of selamectin and doramectin [55].

The competitive inhibition of *Hco-pgp-9.1* transport activity by IVM indicates that it is a substrate of the transporter and that *H. contortus* can partially achieve ML resistance by constitutive higher expression of this transporter, which has been shown to be overexpressed in resistant isolates in different studies. Williamson et al. [11] observed a significant increase of *Hco-pgp-9* mRNA in IVM, BZ, and levamisole triple-resistant L3 from a field isolate. In another study, *Hco-pgp-9.*1 and *Hco-pgp-9.2* showed significantly higher transcript levels in a resistant Wallangra isolate of *H. contortus* than the susceptible control isolate [98]. Kellerova et al. [10] also demonstrated constitutively higher expression of *Hco-pgp-9.1* in the IVM-susceptible *H. contortus* exposed to IVM for 4, 12 and 24 h.

Expression of orthologs of *Hco-pgp-9.1* in other nematodes such as *C. elegans*, *T. circumcincta*, and *P. univalens* further suggest the importance of *Hco-pgp-9.1* in ML resistance. Gerhard et al. [58] showed that the overexpression of *P. univalens Pun-pgp-9* in *C. elegans* influences IVM and MOX susceptibility, and proposed that ML tolerance is regulated in a tissue-specific manner. They demonstrated that intestinal *Pun-pgp-9* expression elicited a protective effect against IVM, while MOX susceptibility was minimally affected when the drug was actively ingested by pharyngeal pumping. Conversely, epidermal *Pun-pgp-9* protected against MOX regardless of pharyngeal pumping activity. This finding might be attributable to the ability of PGPs to prevent substrates from entering the cytosol, and thus represent a protective mechanism (vacuum cleaner model) against potentially toxic molecules [101]. In contrast, protection against IVM has only been shown in the absence of active drug ingestion [58]. In a ML-suceptible *H. contortus* isolate, expression of *pgp-9.1* was shown by immunolocalization to be primarily localized in the uterus of female worms [63]. Epidermal and/or intestinal *pgp-9.1* overexpression contributing to ML susceptibility in the resistant BSI isolate examined here cannot be excluded. Whether *Hco-pgp-9.1* also exhibits a tissue-specific ML protection mechanism needs to be evaluated in future studies.

In contrast to the results presented here, for *Hco-pgp-9.1*, other studies also reported overexpression of *Hco-pgp-10* [10], *Hco-pgp-11* [64, 98], *Hco-pgp-16* [99] and *Hco-pgp-13* [10] in various resistant *H. contortus* isolates as well as in other nematodes, such as *C. elegans* [29, 49, 50] and *P. equorum* [59, 102]. Differences in constitutive expression of *Hco-pgp-10, Hco-pgp-11*, and *Hco-pgp-16* among studies might result from experimental differences, including the developmental stage used, isolate genomic background, drug concentration, incubation time, and in vivo and in vitro conditions. While our study utilized L4, studies referenced by Laing et al. [64] and Kellerova et al. [10] reported comparative analyses for adult stages. The fact that these experimental differences did not seem to affect the results for *Hco-pgp-9.1* may further indicate the particular importance of this PGP in ML resistance. At the same time, other PGPs, such as *pgp-10*, *pgp-11*, and *pgp-16*, probably contribute to ML defense to varying degrees, at least in some isolates or developmental stages.

The downregulation or repression of transporter genes to decrease the number of transporter proteins actively transporting drugs into the tissues of organisms has also been described [103, 104]. The present study only revealed downregulation of *Hco-pgp-13* at 6 and 12 h upon exposure to IVM and at 12 h for MOX.

In silico docking and IVM binding affinity of *Hco-pgp-13* predicted a high affinity to the protein's inner chamber [12], which can result in inhibition of the transport mechanism of *Hco-pgp-13*. Moreover, the same study identified *Hco-pgp-13* localization in epithelial, pharyngeal, intestinal, and neuronal tissues [12]. In particular, PGP expression in the neurons for the transport of endogenous substrates such as lipids by the flippase model can play a role in the access of MLs to targets and lead to a lethal anthelmintic effect [12, 85, 101, 105]. Hence, constitutively lower expression of *Hco-pgp-13* in the BSI isolate may contribute to a protective effect rather than resistance, by minimizing ML transport into the target tissue, and thereby preventing ML-induced hyperpolarization of the neurons and muscle paralysis.

The constitutively lower expression was even enhanced by drug exposure in a time-dependent manner, with differences observed for IVM and MOX, potentially minimizing further influx of MLs into particular tissues of the organism. The delayed onset of *Hco-pgp-13* downregulation with MOX at 12 h also supports differences in IVM and MOX protection mechanisms due to pharmacokinetic differences [40–42]. Tissue-specific and spatial expression levels were not investigated, but might provide further information on the observed differences between gene expression for IVM at 6 h compared to MOX at 12 h in this experimental setting.

Another ABC transporter superfamily member that demonstrated significant upregulation in response to MOX was *Hco-mrp-5*. The differential gene expression analysis showed *Hco-mrp-5* inducibility at 12 h upon MOX exposure and no IVM-dependent effect. In *C. elegans*, *mrp-1* and *mrp-4* were constitutively highly expressed in an IVM-resistant strain relative to the wild-type [106], while *mrp-3* and *mrp-6* were constitutively highly expressed in IVM- and MOX-selected *C. elegans* strains [29]. Similarly, *C. oncophora* recovered from animals treated with IVM exhibited overexpression of *mrp-1* [56, 57].

## Conclusions

This study confirms variability in the constitutive expression of individual CYPs and ABC transporter proteins in a ML-resistant *H. contortus* isolate compared to a susceptible isolate. Our findings align with previous studies that showed that ML resistance is multigenetic [107, 108], and that multiple mechanisms, such as constitutively higher expression of *cyp* genes and ABC transporters, contribute to resistance rather than dominant, single genes necessarily driving resistance. However, even though no IVM- and MOX-inducible *cyp* gene expression was observed, drug exposure led to downregulation of *Hco-pgp-13* at different time points for IVM and MOX, as well as MOX-specific upregulation of *Hco-mrp-5*. This suggests that, although inducible gene expression upon drug exposure occurred, it played only a minor role in resistance in the present study. We therefore conclude that neither *cyp* nor ABC transporter genes appear to be the main drivers of resistance in the isolate examined here, but can contribute to this in other isolates and free-living nematodes. The slight differences observed in inducible transcription between IVM and MOX were likely due to differences in pharmacological properties of the drugs. The findings reported here suggest that the examined genes are not useful markers for the detection of ML resistance.

## Supplementary Information

Additional file 1.

## Data Availability

All raw data have been deposited in the Sequence Read Archive (formerly known as the Short Read Archive) under the Bioproject accession number PRJNA1105765.
