# Supplementary material for: Differences in constitutive gene expression of cytochrome P450 enzymes and ATP-binding cassette transporter gene expression between a susceptible and a highly macrocyclic lactone-resistant Haemonchus contortus isolate in the absence of drug-inducible expression
Source: Parasit Vectors. 2024 Dec 12;17:505. doi: 10.1186/s13071-024-06568-z (PMC11636055; doi:10.1186/s13071-024-06568-z)
Supplement: Supplementary file 1 — Additional file 1. [file 13071_2024_6568_MOESM1_ESM.pdf]

Supplementary File 1

Differences in constitutive gene expression of Cytochrome P450 enzyme and ABC transporter gene expression between a susceptible and a highly macrocyclic-lactone resistant Haemonchus contortus isolate in the absence of drug inducible expression

Natalie Jakobs[1], Sandro Andreotti[2], Sabrina Ramünke[1], Georg von Samson-Himmelstjerna[1], Jürgen Krücken[1,3]

[1] Institute for Parasitology and Tropical Veterinary Medicine, Freie Universität Berlin, Berlin, Germany

[2] Institute of Computer Science, Bioinformatics Solution Center, Freie Universität Berlin, Berlin, Germany

Table S1 Summary of *Ascaris suum* putative cytochrome P450 (CYP) sequences and prediction of CYP heme-iron ligand motifs. All sequences were identified within the *A. suum* draft genome assembly (PRJNA62057) (Wang et al. (2017)).

Gene model number[a]	Gene transcript	Number of amino acids[b]	Cytochrome P450 cysteine heme-iron ligand motif[c,d]		Note
AgR060_g025	AgR060_g025_t01	1348	1287 – 1296	FGiGKRQCAG	
AgB05_g394	AgB05_g394_t04	562	495 – 504	FSiGKRQCLG	
AgB04_g011	AgB04_g011_t01	453	440 – 449	FSaGSRNCIG	
AgB02_g153	AgB02_g153_t01	357	293 – 302	FSaGPRNCIG	5'
AgR071_g004	AgR071_g004_t01	536	472 – 481	FGiGPRNCIG	5'
AgB02_g399	AgB02_g399_t01	509	449 – 458	FSaGIRNCIG	
AgR026_g126	AgR026_g126_t01	505	441 – 450	FSaGPRNCIG	
AgR003_g350	AgR003_g350_t04	457	395 – 404	FGaGPRMCIG	
AgR005_g136	AgR005_g136_t01	536	472 – 481	FGfGPRNCIG	
AgR003_g350	AgR003_g350_t04	457	395 – 404	FGaGPRMCIG	
AgR008_g167	AgR008_g167_t03	528	467 – 476	FGaGPRQCIG	

[a] Gene model number corresponding to *A. suum* genome assembly PRJNA62057 (Wang et al. (2017)).
[b] Predicted by ExPASy – ProtParam (https://web.expasy.org/protparam/).
[c] Predicted by ExPASy – ScanProsite (https://prosite.expasy.org/scanprosite/).
[d] Motif position within the amino acid sequence and sequence motif.
[e] 5': 5'-end of the peptide sequence was manually corrected.

Table S2 Summary of *Parascaris univalens* putative cytochrome P450 (CYP) sequences and prediction of CYP heme-iron ligand motifs. All sequences were identified within the *P. univalens* genome assembly (PRJNA386823) (Wang et al. (2017)).

Gene model number[a]	Gene transcript	Number of amino acids[b]	Cytochrome P450 cysteine heme-iron ligand motif[c,d]		Note
PgR002_g027	PgR002_g027_t02	518	457 – 466	FGaGPRQCIG	
PgR010_g185	PgR010_g185_t02	536	472 – 481	FGfGPRNCIG	
PgR114_g002	PgR114_g002_t01	536	472 – 481	FGiGPRNCIG	5'
PgR020_g037	PgR020_g037_t01	493	432 – 441	FGiGKRQCAG	
PgR006_g150	PgR006_g150_t01	509	440 – 449	FSaGSRNCIG	
PgR071_g005	PgR071_g005_t01	505	441 – 450	FSaGPRNCIG	
PgR027_g023	PgR027_g023_t01	400	360 – 369	FGfGKRSCLG	
PgB17_g019	PgB17_g019_t01	379	310 – 319	FSaGPRNCLG	5'
PgR006_g150	PgR006_g150_t01	509	440 – 449	FSaGSRNCIG	
PgR033_g054	PgR033_g054_t01	438	378 – 387	FSaGIRNCIG	

[a] Gene model numbers corresponding to *P. univalens* genome assembly PRJNA386823 (Wang et. al. (2017)).

[b] Predicted by ExPASy – ProtParam (https://web.expasy.org/protparam/).

[c] Predicted by ExPASy – ScanProsite (https://prosite.expasy.org/scanprosite/).

[d] Motif position within the amino acid sequence and sequence motif.

[e] 5': 5'-end of the peptide sequence was manually corrected.

Table S3 Summary of putative *Haemonchus contortus* cytochrome P450 (CYP) sequences identified by Laing et al. (2015) and prediction of Cyp heme-iron ligand motifs. Peptide sequences of gene model numbers by Laing et al. (2015) were used in a tBLASTn search to identify gene model numbers in the corresponding *H. contortus* draft genome assembly PRJEB506 (Doyle et al. (2020)).

Putative *H. contortus* CYPs identified by Laing et al. (2015)		Number of amino acids[a]	Cytochrome P450 cysteine heme-iron ligand motif[b,c]		Gene model number corresponding to PRJEB506(50)
GenBank Accession number	Gene model numbers based on Laing et al. (2013) (61)				
CDJ81012.1	HCOI_00255000	505	447 – 456	FGfGPRMCAG	HCON_00044070
CDJ82292.1	HCOI_00284400	515	452 – 461	FGlGPRQCLG	HCON_00141052
CDJ82330.1	HCOI_00382500	985	494 – 503	FGmGKRTCLG	HCON_00038080
CDJ83273.1	HCOI_00383400	500	437 – 446	FGlGKRSCLG	HCON_00022640
CDJ83276.1	HCOI_00383700	499	437 – 446	FGiGRRACLG	HCON_00022670
CDJ84496.1	HCOI_01407900	444	413 – 422	FSiGKRQCLG	HCON_00145170
CDJ84541.1	HCOI_00653300	512	442 – 451	FSaGSRNCIG	HCON_00145460
CDJ84895.1	HCOI_00576400	145	80 – 89	FGlGKRSCIG	HCON_00073880
CDJ86031.1	HCOI_00816200	378	316 – 325	FSiGKRQCLG	HCON_00143950
CDJ87159.1	HCOI_00827700	1111	1048 – 1057	FGlGPRQCIG	HCON_00141052
CDJ92488.1	HCOI_01579500	250			HCON_00038960
CDJ92489.1	HCOI_01579600	225	152 – 161	FSaGSRNCIG	HCON_00038960
CDJ94176.1	HCOI_01920700	196			HCON_00024010
CDJ94556.1	HCOI_01637300	213	153 – 162	FSaGPRNCIG	HCON_00136335
CDJ95149.1	HCOI_01704800	188			HCON_00134870
CDJ96396.1	HCOI_02017000	443	382 – 391	FGlGPRQCVG	HCON_00084620
CDJ96745.1	HCOI_02053000	493	432 – 441	FSmGKRQCLG	HCON_00133050
CDJ97632.1	HCOI_02145700	493	430 – 439	FSvGKRACLG	HCON_00030320
CDJ97895.1	HCOI_02168300	466	393 – 402	FSvGKRQCLG	HCON_00042000
CDJ97964.1	HCOI_01928800	928	842 – 851	FGiGKRSCLG	HCON_00073880
CDJ97972.1	HCOI_01929700	928	842 – 851	FGiGKRSCLG	HCON_00024010
CDJ98403.1	HCOI_00165400	270	210 – 219	FSaGPRNCIG	HCON_00045460
CDJ98491.1	HCOI_00046100	409			HCON_00117780
	HCOI_01487700	326	253 – 262	FSvGKRQCLG	HCON_00042000

[a] Predicted by ExPASy – ProtParam (https://web.expasy.org/protparam/).

[b] Predicted by ExPASy – ScanProsite (https://prosite.expasy.org/scanprosite/).

[c] Motif position within the amino acid sequence and sequence motif.

Table S4 Summary of *Haemonchus contortus* putative cytochrome P450 sequences and prediction of protein parameters. All sequences were identified using the *H. contortus* draft genome assembly (PRJEB506) (Doyle et al. (2020)).

Gene model IDs[a]	Number of amino acids[b]	Molecular weight [kDa][b]	Theoretical pI[b]	Cytochrome P450 cysteine heme-iron ligand motif[c,d]	
HCON_00030320	493	57.69	6.72	430 – 439	FSvGKRACLG
HCON_00042000	510	58.85	6.58	436 – 445	FSvGKRQCLG
HCON_00084620	443	51.21	8.71	382 – 391	FGlGPRQCVG
HCON_00141052	517	59.55	8.76	454 – 463	FGlGPRQCIG
HCON_00038960	502	57.93	7.54	429 – 438	FSaGSRNCIG
HCON_00143950	494	57.34	5.93	432 – 441	FSiGKRQCLG
HCON_00045460	499	57.42	6.90	439 – 448	FSaGPRNCI
HCON_00022640	500	58.14	8.42	437 – 446	FGlGKRSCLG
HCON_00022670-2	499	56.92	6.48	437 – 446	FGiGRRACLG
HCON_00133050	458	53.01	6.10	397 – 406	FSmGKRQCLG
HCON_00136335	508	59.04	6.55	448 – 457	FSaGPRNCIG
HCON_00145170	446	52.02	5.99	413 – 422	FSiGKRQCLG
HCON_00145460	512	58.83	7.98	442 – 451	FSaGSRNCIG
HCON_00023990	495	57.37	7.94	433 – 442[f]	FPLDEKQCTR[f]
HCON_00024020	492	56.20	8.09	432 – 441	FSiGKRMCVG
HCON_00024010	511	58.26	8.56	451 – 460	FSiGKRMCVG
HCON_00117780	541	61.49	7.85	480 – 489	FGyGPRNCIG
HCON_00024000	495	56.56	6.91	433 – 442	FSmGKRQCAG
HCON_00024005	468	53.64	7.62	406 – 415	FSmGKRQCAG
HCON_00134870	425	48.47	5.58	378 – 387	WSaGPRNCIG
HCON_00038080	532	61.26	8.00	465 – 474	FGmGKRTCLG
HCON_00073880	504	58.45	7.25	439 – 448	FGlGKRSCIG
HCON_00073890	503	58.46	6.73	439 – 448	FGiGKRSCLG
HCON_00044070	505	57.74	9.02	447 – 456	FGfGPRMCAG
HCON_00038090	520	58.95	8.20	425 – 434[d]	HDYADEACLM[d]

[a] Gene model IDs corresponding to *Haemonchus contortus* genome assembly PRJEB506 (Doyle et al. (2020)).

[b] Predicted by ExPASy – ProtParam (https://web.expasy.org/protparam/).

[c] Predicted by ExPASy – ScanProsite (https://prosite.expasy.org/scanprosite/).

[d] Motif position within the amino acid sequence and sequence motif.

[f] Identified via sequence alignment with HCON_00141052

Table S5 Differential gene expression analysis of *Haemonchus contortus* cytochrome P450 enzymes in fourth-stage larvae from an ivermectin & moxidectin-resistant *H. contortus* berlin-selected isolate (BSI) vs. drug-susceptible *H. contortus* McMaster (McM). Samples were obtained from in vitro culture with larvae exposed to 0.05% DMSO for 3 h. Mean normalized counts (DESeq2), obtained from RNA sequencing, were used to calculate $\log_2$ fold-changes ($\log_2$FC) between *H. contortus* BSI vs. *H. contortus* McM. P-values were determined by Mann Whitney test: ***, p-value < 0.001; **, p-value < 0.01; *, p-value < 0.05.

Gene model ID[a]	$\log_2$ Fold-Change (*Hc* BSI vs. *Hc* McM DMSO 3 hours)	*p-value*	
Hco-cyp-13			
HCON_00141052	0,68	0,0446	*
Hco-cyp-14			
HCON_00024000	1,01	0,0050	**
HCON_00024005	1,30	0,0022	**
HCON_00024010	1,48	0,0022	**
HCON_00024020	-1,06	0,0022	**
HCON_00023990	0,92	0,0022	**
Hco-cyp-23			
HCON_00042000	0,51	0,0049	**
Hco-cyp-25			
HCON_00117780	0,09	0,8726	
Hcp-cyp-31			
HCON_00038090	0,54	0,4641	
Hco-cyp-32			
HCON_00136335	-1,13	0,0050	**
HCON_00134870	1,46	0,0048	**
Hco-cyp-33			
HCON_00145170	0,70	0,0022	**
HCON_00143950	-0,39	0,0649	
HCON_00133050	0,09	0,3939	
Hco-cyp-34/35			
HCON_00073880	0,66	0,0022	**
HCON_00022670	0,17	0,3939	
HCON_00022640	-0,44	0,0022	**
HCON_00073890	1,17	0,0050	**
Hco-cyp-36			
HCON_00030320	0,05	0,4225	
HCON_00038080	-0,07	0,4848	
Hco-cyp-37			
HCON_00045460	-0,23	0,5887	
Hco-cyp-39			
HCON_00038960	-0,30	0,3095	
Hco-cyp-42			
HCON_00145460	0,93	0,0022	**
Hco-cyp-43			
HCON_00084620	0,04	0,4704	
Hco-cyp-44			
HCON_00044070	1,41	0,0022	**

[a] Gene model IDs corresponding to *Haemonchus contortus* genome assembly PRJEB506 (Doyle et al. (2020)).

Table S6 Time course of relative basal expression (mean normalized counts and standard error of mean (SEM)) of cytochrome P450 enzyme transcripts in *Haemonchus contortus* berlin-selected isolate (BSI) fourth-stage larvae. The data were obtained from six biological replicates per isolate and exposed to 0.05% DMSO for 3, 6, and 12 hours. P-values were determined by Kruskal-Wallis test with Dunn's post-hoc test.

Gene model number[a]	3 hours		6 hours		12 hours		*p-value*
	Mean	SEM	Mean	SEM	Mean	SEM	
Hco-cyp-13							
HCON_00141052	109,08	14,14	132,55	14,42	145,30	7,44	0,3679
Hco-cyp-14							
HCON_00024000	322,91	23,36	322,85	24,48	353,70	9,90	0,3679
HCON_00024005	725,16	56,10	760,29	78,19	731,48	66,91	0,3679
HCON_00024010	680,43	68,33	788,58	112,22	882,72	80,79	0,3679
HCON_00024020	504,56	32,76	552,06	43,56	575,72	24,36	0,3679
HCON_00023990	92,85	7,76	96,12	11,37	91,58	5,93	0,3679
Hco-cyp-23							
HCON_00042000	352,75	25,01	312,91	21,52	263,66	20,20	0,3679
Hco-cyp-25							
HCON_00117780	104,21	5,24	92,89	3,81	99,85	3,55	0,3679
Hcp-cyp-31							
HCON_00038090	3,52	0,83	3,41	0,72	3,89	0,86	0,3679
Hco-cyp-32							
HCON_00136335	64,10	4,41	71,14	8,23	81,17	8,34	0,3679
HCON_00134870	216,68	19,91	223,43	17,68	199,38	11,35	0,3679
Hco-cyp-33							
HCON_00145170	149,68	10,70	146,47	13,14	168,34	11,86	0,3679
HCON_00143950	127,80	9,29	134,31	6,85	123,80	2,09	0,3679
HCON_00133050	530,92	13,59	486,55	19,16	472,88	16,45	0,3679
Hco-cyp-34/35							
HCON_00073880	176,51	11,90	165,72	13,58	186,60	12,37	0,3679
HCON_00022670	108,11	7,85	108,78	5,95	112,54	5,81	0,3679
HCON_00022640	521,27	10,12	498,69	17,79	474,60	12,17	0,3679
HCON_00073890	335,68	28,36	323,64	26,88	316,40	14,93	0,3679
Hco-cyp-36							
HCON_00030320	21,54	2,09	19,93	2,07	22,66	3,46	0,3679
HCON_00038080	914,80	84,38	897,75	80,82	721,99	24,85	0,3679
Hco-cyp-37							
HCON_00045460	86,39	1,65	79,89	3,73	95,64	8,19	0,3679
Hco-cyp-39							
HCON_00038960	50,09	2,35	42,42	2,66	43,99	2,64	0,3679
Hco-cyp-42							
HCON_00145460	1940,92	167,67	1721,46	154,41	1577,74	118,74	0,3679
Hco-cyp-43							
HCON_00084620	78,27	5,83	72,90	3,43	75,73	3,91	0,3679
Hco-cyp-44							
HCON_00044070	237,52	40,38	230,01	37,38	172,50	15,28	0,3679

[a] Gene model IDs corresponding to *Haemonchus contortus* genome assembly PRJEB506 (Doyle et al. (2020)).

Table S7 Differential gene expression analysis of *Haemonchus contortus* cytochrome P450 enzymes in *H. contortus* berlin-selected isolate (BSI) fourth-stage larvae upon exposure to 100 nM ivermectin (IVM), 100 nM moxidectin (MOX) and 0.05% DMSO (vehicle control). Samples were obtained from in vitro culture with larvae exposed for 3 h, 6 h, and 12 h. Mean normalized counts (DESeq2), obtained from RNA sequencing, were used to calculate log_2 fold-changes (log_2FC) between IVM and MOX-treated *H. contortus* vs. DMSO-treated *H. contortus*.

Gene model number	DMSO			Ivermectin			Moxidectin		
	3 hours	6 hours	12 hours	3 hours	6 hours	12 hours	3 hours	6 hours	12 hours
Hco-cyp-13									
HCON_00141052	-0,06	0,24	0,40	0,12	0,06	0,20	0,11	0,06	0,12
Hco-cyp-14									
HCON_00024000	-0,02	-0,02	0,13	-0,13	-0,16	-0,05	-0,07	-0,09	0,01
HCON_00024005	-0,02	0,03	-0,02	0,10	0,07	0,13	0,15	0,16	0,13
HCON_00024010	-0,03	0,16	0,35	0,00	0,15	0,31	0,17	0,26	0,29
HCON_00024020	-0,02	0,11	0,18	0,14	0,14	0,17	0,14	0,22	0,19
HCON_00023990	-0,02	0,00	-0,03	-0,14	-0,19	-0,13	-0,07	-0,21	-0,01
Hco-cyp-23									
HCON_00042000	-0,02	-0,19	-0,44	-0,12	-0,09	-0,25	-0,35	-0,06	-0,21
Hco-cyp-25									
HCON_00117780	-0,01	-0,17	-0,07	-0,33	-0,21	-0,16	-0,04	-0,15	-0,10
Hcp-cyp-31									
HCON_00038090	-0,23	-0,28	-0,07	0,78	-0,12	0,43	-0,18	-0,50	0,50
Hco-cyp-32									
HCON_00136335	-0,02	0,10	0,29	-0,05	-0,10	-0,02	-0,05	-0,18	0,03
HCON_00134870	-0,03	0,02	-0,13	-0,11	0,08	-0,02	-0,02	0,04	-0,02
Hco-cyp-33									
HCON_00145170	-0,02	-0,06	0,15	0,06	0,15	0,07	0,07	0,16	0,11
HCON_00143950	-0,02	0,06	-0,05	0,09	-0,11	-0,26	0,03	-0,11	-0,06
HCON_00133050	0,00	-0,13	-0,17	-0,05	-0,17	-0,19	-0,12	-0,17	-0,12
Hco-cyp-34/35									
HCON_00073880	-0,02	-0,12	0,06	-0,14	-0,08	-0,09	-0,25	-0,07	-0,01
HCON_00022670	-0,02	0,00	0,05	-0,10	-0,01	0,10	0,07	-0,15	0,03
HCON_00022640	0,00	-0,07	-0,14	0,10	-0,01	0,01	0,09	0,05	0,06
HCON_00073890	-0,03	-0,08	-0,09	-0,24	-0,09	-0,07	-0,24	-0,16	0,02
Hco-cyp-36									
HCON_00030320	-0,04	-0,15	-0,02	0,37	-0,41	0,15	0,03	-0,33	0,06
HCON_00038080	-0,03	-0,06	-0,35	-0,05	-0,07	-0,13	-0,18	-0,08	-0,06
Hco-cyp-37									
HCON_00045460	0,00	-0,12	0,12	0,01	0,05	0,27	0,25	0,01	0,21
Hco-cyp-39									
HCON_00038960	-0,01	-0,25	-0,20	0,08	-0,17	-0,01	-0,17	-0,39	-0,08
Hco-cyp-42									
HCON_00145460	-0,03	-0,20	-0,32	-0,10	-0,18	-0,22	-0,21	-0,18	-0,18
Hco-cyp-43									
HCON_00084620	-0,02	-0,11	-0,06	-0,16	-0,22	-0,24	-0,13	-0,09	-0,29
Hco-cyp-44									
HCON_00044070	-0,09	-0,14	-0,49	-0,08	-0,04	-0,09	-0,19	-0,07	0,11

Table S8 Differential gene expression analysis of *Haemonchus contortus* ABC transporter in fourth-stage larvae from an ivermectin & moxidectin-resistant *H. contortus* berlin-selected isolate (BSI) vs. drug-susceptible *H. contortus* McMaster (McM). Samples were obtained from in vitro culture with larvae exposed to 0.05% DMSO for 3 h. Mean normalized counts (DESeq2), obtained from RNA sequencing, were used to calculate $\log_2$ fold-changes ($\log_2$FC) between *H. contortus* BSI vs. *H. contortus* McM. P-values were determined by Mann Whitney test: ***, p-value < 0.001; **, p-value < 0.01; *, p-value < 0.05.

Gene model number[a]	Gene name[b]	$\log_2$ Fold-Change (*Hc* BSI vs. *Hc* McM DMSO 3 hours)	*p-value*	
HCON_00092830	abce-1	0,05	0,4848	
HCON_00150870	abcf-1	-0,41	0,0022	**
HCON_00090460	abcf-3	-0,10	0,4848	
HCON_00035372	abch-1	0,71	0,0048	**
HCON_00035374	abcx-1	0,84	0,0649	
HCON_00030810	abt-2	0,00	0,8182	
HCON_00046480	abt2b	-0,15	0,6991	
HCON_00092350	ced-7a	0,38	0,0152	*
HCON_00094190	ced-7b	-0,22	0,1320	
HCON_00094200	ced-7d	-0,32	0,0931	
HCON_00094310	ced-7e	-0,04	0,9372	
HCON_00193610	ced-7f	-0,05	0,8182	
HCON_00085890	abt-4	0,33	0,0649	
HCON_00037705	abt-7	0,37	0,1320	
HCON_00066100	abt-8	0,34	0,2946	
HCON_00011130	abtm-1	-0,14	0,3939	
HCON_00140610	haf-3.1	-0,29	0,0152	*
HCON_00018690	haf-4	0,29	0,0651	
HCON_00126030	haf-6	-0,11	0,5887	
HCON_00003100	haf-9	0,07	0,3095	
HCON_00075740	hmt-1	-0,13	0,9372	
HCON_00189480	mrp-1	-0,12	0,5887	
HCON_00175410	mrp-4	-0,68	0,0022	**
HCON_00164880	mrp-5	-0,15	0,3939	
HCON_00110560	mrp-7	-0,04	0,8182	
HCON_00145390	cft-1	0,89	0,0050	**
HCON_00144960	mrp-6	-0,18	0,3095	
HCON_00098130	pgp-1	-0,34	0,0931	
HCON_00004450	pgp-2	-0,22	0,1320	
HCON_00042800	pgp-3	-1,17	0,0022	**
HCON_00035895	pgp-16	-1,05	0,0022	**
HCON_00130050	pgp-9.1	0,49	0,0152	*
HCON_00130060	pgp-9.2	-0,10	0,6991	
HCON_00168800	pgp-10	-0,44	0,0022	**
HCON_00054780	pgp-17	1,30	0,0526	
HCON_00162780	pgp-11	-1,17	0,0022	**
HCON_00041390	pgp-13	-0,38	0,0247	*
HCON_00053980	pmp-2	-0,41	0,0043	**
HCON_00135800	pmp-3	-0,15	0,4848	
HCON_00099610	pmp-4	-0,31	0,0649	
HCON_00063000	pmp-5	-0,64	0,0022	**
HCON_00187360	pmp-6.1	0,01	0,8182	
HCON_00129680	pmp-7	-0,56	0,0777	
HCON_00067980	wht-1	0,45	0,0651	
HCON_00112160	wht-2	-0,25	0,0542	
HCON_00060120	wht-4	0,43	0,0260	*
HCON_00065970	wht-5a	-0,44	0,0411	*
HCON_00065975	wht-5b	-0,26	0,0649	
HCON_00068420	wht-7	-0,57	0,0043	**
HCON_00116670	wht-8	2,37	0,0047	**

[a] Gene model IDs corresponding to *Haemonchus contortus* genome assembly PRJEB506 (Doyle et. al (2020)) and selected from Mate et al. (2022).

[b] *Haemonchus contortus* proposed gene name based on Mate et al. (2022).

Table S9 Time course of relative basal expression (mean normalized counts and standard error of mean (SEM)) of ABC transporter transcripts in *Haemonchus contortus* berlin-selected isolate (BSI) fourth-stage larvae. The data were obtained from six biological replicates per isolate and exposed to 0.05% DMSO for 3, 6, and 12 hours. P-values were determined by Kruskal-Wallis test with Dunn's post-hoc test: ***, p-value < 0.001; **, p-value < 0.01; *, p-value < 0.05.

Gene model number[a]	Gene name[b]	3 hours		6 hours		12 hours		*p-value*
		Mean	SEM	Mean	SEM	Mean	SEM	
HCON_00092830	abce-1	3077,97	75,07	3201,93	30,96	3338,32	69,37	0,3679
HCON_00150870	abcf-1	4201,14	51,52	4568,22	90,30	4899,69	98,41	0,3679
HCON_00090460	abcf-3	4829,33	125,75	5274,07	64,77	5371,34	75,75	0,3679
HCON_00035372	abch-1	20,99	2,16	24,83	1,30	30,07	1,99	0,3679
HCON_00035374	abcx-1	48,36	5,74	45,34	4,55	44,59	3,62	0,3679
HCON_00030810	abt-2	2744,33	48,11	2914,61	59,09	3036,65	41,75	0,3679
HCON_00046480	abt2b	1795,70	104,55	1830,31	81,07	2038,39	50,30	0,3679
HCON_00092350	ced-7a	278,57	16,11	281,42	11,95	288,29	4,72	0,3679
HCON_00094190	ced-7b	5351,60	118,38	5585,97	90,33	5631,11	53,85	0,3679
HCON_00094200	ced-7d	506,08	30,36	507,02	29,52	526,46	26,34	0,3679
HCON_00094310	ced-7e	211,57	8,40	230,40	13,78	247,54	8,56	0,3679
HCON_00193610	ced-7f	586,71	22,59	529,85	21,24	515,17	16,73	0,3679
HCON_00085890	abt-4	1180,98	65,95	1218,22	50,74	1228,10	23,08	0,3679
HCON_00037705	abt-7	115,79	5,91	105,22	13,79	107,50	12,97	0,3679
HCON_00066100	abt-8	22,23	2,05	23,79	1,77	18,48	1,93	0,3679
HCON_00011130	abtm-1	5668,07	154,94	5572,52	117,00	5589,04	77,95	0,3679
HCON_00140610	haf-3.1	1784,53	33,34	1795,49	32,58	1792,10	37,25	0,3679
HCON_00018690	haf-4	37,23	3,49	44,95	2,52	42,08	3,37	0,3679
HCON_00126030	haf-6	2061,57	48,52	2076,49	43,01	2176,70	57,11	0,3679
HCON_00003100	haf-9	277,77	15,62	298,73	11,33	311,01	11,96	0,3679
HCON_00075740	hmt-1	1846,81	43,31	1858,69	25,26	1938,83	41,15	0,3679
HCON_00189480	mrp-1	4847,32	145,48	5279,46	128,54	5764,62	94,86	0,3679
HCON_00175410	mrp-4	267,79	16,91	239,41	14,60	236,86	9,77	0,3679
HCON_00164880	mrp-5	4989,30	107,26	4850,39	128,49	4713,01	34,06	0,3679
HCON_00110560	mrp-7	1183,88	36,69	1187,32	37,58	1224,91	34,06	0,3679
HCON_00145390	cft-1	378,37	8,79	370,17	17,79	358,71	12,67	0,3679
HCON_00144960	mrp-6	2803,35	25,06	2852,71	42,61	2876,50	37,98	0,3679
HCON_00098130	pgp-1	1304,91	76,86	1354,41	82,80	1344,26	39,15	0,3679
HCON_00004450	pgp-2	714,57	18,77	768,88	22,79	771,57	23,86	0,3679
HCON_00042800	pgp-3	855,10	62,35	940,45	59,68	906,73	32,57	0,3679
HCON_00035895	pgp-16	321,01	25,02	331,78	26,42	346,24	10,79	0,3679
HCON_00130050	pgp-9.1	5118,88	321,57	5807,17	501,46	6874,65	397,20	0,3679
HCON_00130060	pgp-9.2	2722,88	219,30	2950,91	247,25	3414,69	159,60	0,3679
HCON_00168800	pgp-10	3165,76	149,83	3070,02	134,32	2934,12	104,46	0,3679
HCON_00054780	pgp-17	8,34	1,53	8,39	2,79	7,01	1,60	0,3679
HCON_00162780	pgp-11	188,12	9,66	194,99	9,07	204,50	5,87	0,3679
HCON_00041390	pgp-13	129,38	5,52	133,03	8,74	159,26	10,72	0,3679
HCON_00053980	pmp-2	567,38	16,09	517,68	21,29	465,01	12,68	0,3679
HCON_00135800	pmp-3	1792,27	11,68	1773,49	45,48	1771,10	40,33	0,3679
HCON_00099610	pmp-4	507,67	38,50	508,27	28,99	472,67	18,85	0,3679
HCON_00063000	pmp-5	1031,00	38,05	1028,63	45,73	1043,61	31,26	0,3679
HCON_00187360	pmp-6.1	493,04	25,24	532,67	27,30	531,10	28,61	0,3679
HCON_00129680	pmp-7	22,32	2,51	26,12	2,09	26,33	4,41	0,3679
HCON_00067980	wht-1	90,30	5,15	89,46	5,21	95,55	5,90	0,3679
HCON_00112160	wht-2	455,92	13,40	488,32	14,75	499,57	15,66	0,3679
HCON_00060120	wht-4	48,78	3,44	39,35	3,15	37,07	2,31	0,3679
HCON_00065970	wht-5a	342,57	22,13	341,22	7,86	338,85	11,03	0,3679
HCON_00065975	wht-5b	409,10	14,40	354,47	11,13	382,41	10,80	0,3679
HCON_00068420	wht-7	255,40	12,15	254,72	7,63	247,54	7,89	0,3679
HCON_00116670	wht-8	19,53	2,22	30,88	4,59	33,64	3,16	0,3679

[a] Gene model IDs corresponding to *Haemonchus contortus* genome assembly PRJEB506 (Doyle et al. (2020)) and selected from Mate et al. (2022).

[b] *Haemonchus contortus* proposed gene name based on Mate et al. (2022).

Table S8 Differential gene expression analysis of *Haemonchus contortus* ABC transporter in *H. contortus* BSI fourth-stage larvae upon exposure to 100 nM ivermectin, 100 nM moxidectin and 0.05% DMSO (vehicle control). Samples were obtained from in vitro culture with larvae exposed for 3 h, 6 h, and 12 h. Mean normalized counts (DESeq2), obtained from RNA sequencing, were used to calculate log_2 fold-changes (log_2FC) between IVM and MOX-treated *H. contortus* vs. DMSO-treated *H. contortus*.

Gene model number[a]	Gene name[b]	DMSO			Ivermectin			Moxidectin		
		3 hours	6 hours	12 hours	3 hours	6 hours	12 hours	3 hours	6 hours	12 hours
HCON_00092830	abce-1	0,00	0,06	0,12	0,01	0,04	0,14	0,00	0,06	0,11
HCON_00150870	abcf-1	0,00	0,12	0,22	0,03	0,12	0,23	0,02	0,09	0,17
HCON_00090460	abcf-3	0,00	0,13	0,15	0,01	0,14	0,23	-0,06	0,12	0,21
HCON_00035372	abch-1	-0,04	0,23	0,50	-0,03	0,51	0,10	0,17	0,02	0,39
HCON_00035374	abcx-1	-0,06	-0,13	-0,13	0,02	0,16	0,06	-0,12	0,03	0,16
HCON_00030810	abt-2	0,00	0,09	0,15	0,00	0,08	0,11	0,02	0,11	0,13
HCON_00046480	abt2b	-0,01	0,02	0,18	0,01	0,08	0,20	0,07	0,11	0,23
HCON_00092350	ced-7a	-0,01	0,01	0,05	-0,05	-0,03	0,05	0,04	0,13	0,08
HCON_00094190	ced-7b	0,00	0,06	0,07	0,01	0,04	0,08	0,01	0,03	0,04
HCON_00094200	ced-7d	-0,01	-0,01	0,05	0,00	0,07	0,03	-0,05	0,05	0,13
HCON_00094310	ced-7e	-0,01	0,11	0,22	-0,11	0,24	0,19	0,03	0,10	0,33
HCON_00193610	ced-7f	-0,01	-0,15	-0,19	-0,01	-0,17	-0,24	-0,11	-0,11	-0,16
HCON_00085890	abt-4	-0,01	0,04	0,06	0,04	0,15	0,20	0,15	0,17	0,12
HCON_00037705	abt-7	-0,01	-0,19	-0,11	-0,13	0,12	-0,10	-0,12	0,12	0,10
HCON_00066100	abt-8	-0,03	0,08	-0,28	0,11	-0,05	-0,20	0,28	0,20	-0,20
HCON_00011130	abtm-1	0,00	-0,03	-0,02	-0,03	-0,07	-0,05	0,00	-0,06	-0,04
HCON_00140610	haf-3.1	0,00	0,01	0,00	-0,02	0,00	0,05	-0,01	0,06	0,05
HCON_00018690	haf-4	-0,04	0,26	0,16	0,19	0,13	0,47	0,14	0,31	0,34
HCON_00126030	haf-6	0,00	0,01	0,08	-0,03	0,04	0,04	0,03	0,07	0,03
HCON_00003100	haf-9	-0,01	0,10	0,16	0,06	0,14	0,06	0,13	0,05	0,16
HCON_00075740	hmt-1	0,00	0,01	0,07	0,01	0,07	0,13	0,06	0,10	0,14
HCON_00189480	mrp-1	0,00	0,12	0,25	0,05	0,14	0,25	0,14	0,18	0,26
HCON_00175410	mrp-4	-0,01	-0,18	-0,18	-0,24	-0,11	-0,24	-0,08	-0,04	-0,16
HCON_00164880	mrp-5	0,00	-0,04	-0,08	0,01	0,00	-0,04	0,08	0,04	0,03
HCON_00110560	mrp-7	0,00	0,00	0,05	-0,06	-0,04	0,11	0,06	0,09	0,10
HCON_00145390	cft-1	0,00	-0,04	-0,08	0,12	0,09	-0,05	0,10	0,09	0,00
HCON_00144960	mrp-6	0,00	0,02	0,04	-0,08	0,03	0,06	-0,01	0,04	0,05
HCON_00098130	pgp-1	-0,01	0,04	0,04	0,08	0,04	0,01	0,10	0,13	-0,03
HCON_00004450	pgp-2	0,00	0,10	0,11	-0,04	0,07	0,10	0,00	0,10	0,09
HCON_00042800	pgp-3	-0,02	0,12	0,08	0,09	0,01	-0,05	0,10	0,02	-0,22
HCON_00035895	pgp-16	-0,02	0,03	0,11	-0,11	0,06	-0,03	0,07	0,18	-0,02
HCON_00130050	pgp-9.1	-0,01	0,16	0,42	0,10	0,23	0,48	0,18	0,26	0,40
HCON_00130060	pgp-9.2	-0,02	0,09	0,32	0,11	0,09	0,37	0,17	0,16	0,30
HCON_00168800	pgp-10	-0,01	-0,05	-0,11	0,04	0,00	-0,01	0,08	0,04	0,04
HCON_00054780	pgp-17	-0,21	-0,64	-0,35	-0,34	0,13	0,23	-0,24	-0,25	0,01
HCON_00162780	pgp-11	-0,01	0,04	0,12	-0,12	0,05	-0,04	-0,04	-0,05	-0,10
HCON_00041390	pgp-13	-0,01	0,03	0,28	-0,27	-0,37	-0,05	-0,09	-0,21	-0,25
HCON_00053980	pmp-2	0,00	-0,14	-0,29	0,03	-0,18	-0,19	0,06	0,02	-0,20
HCON_00135800	pmp-3	0,00	-0,02	-0,02	0,02	0,03	0,06	0,00	0,03	0,05
HCON_00099610	pmp-4	-0,02	-0,01	-0,11	0,02	-0,03	-0,09	-0,04	-0,06	-0,11
HCON_00063000	pmp-5	-0,01	-0,01	0,02	0,00	0,01	-0,05	-0,06	-0,03	-0,03
HCON_00187360	pmp-6.1	-0,01	0,10	0,10	-0,02	0,17	0,19	0,06	0,20	0,19
HCON_00129680	pmp-7	-0,04	0,20	0,09	0,03	-0,18	0,05	-0,26	-0,12	-0,31
HCON_00067980	wht-1	-0,01	-0,03	0,07	-0,21	0,02	0,08	-0,01	-0,03	0,11
HCON_00112160	wht-2	0,00	0,10	0,13	0,03	0,04	0,03	0,09	0,00	0,09
HCON_00060120	wht-4	-0,02	-0,33	-0,41	-0,13	0,05	-0,18	0,08	-0,14	-0,20
HCON_00065970	wht-5a	-0,01	-0,01	-0,02	-0,03	0,06	-0,03	0,04	0,06	0,05
HCON_00065975	wht-5b	0,00	-0,21	-0,10	-0,07	0,04	-0,12	0,03	-0,02	-0,02
HCON_00068420	wht-7	-0,01	-0,01	-0,05	-0,01	-0,09	-0,01	-0,05	0,01	-0,03
HCON_00116670	wht-8	-0,06	0,58	0,77	0,33	0,44	0,50	-0,13	-0,06	0,49

[a] Gene model IDs corresponding to *Haemonchus contortus* genome assembly PRJEB506 (Doyle et al. (2020)) and selected from Mate et al. (2022).

[b] Haemonchus contortus proposed gene names based on Mate et al. (2022).

Table S11 Command lines for RNA-Seq data processing tools

Tool	Command
Cutadapt	-a AGATCGGAAGAGCACACGTCTGAACTCCAGTCA -A AGATCGGAAGAGCGTCGTGTAGGGAAAGAGTGT -a A[100] -g T[100] -q 20 -m 36
STAR (index)	--runMode genomeGenerate --sjdbOverhang 149 --sjdbGTFfile haemonchus_contortus.PRJEB506.WBPS15.annotations.gtf
STAR (mapping)	STAR --outSAMtype BAM SortedByCoordinate --quantMode GeneCounts --outFilterType BySJout --outFilterMultimapNmax 20 --alignSJoverhangMin 8 --alignSJDBoverhangMin 1 --outFilterMismatchNmax 999 --outFilterMismatchNoverReadLmax 0.15 --alignIntronMin 20 --alignIntronMax 1000000 --alignMatesGapMax 1000000 --runThreadN 6
FeatureCounts	-s 0 -p -R "BAM" -t “exon"

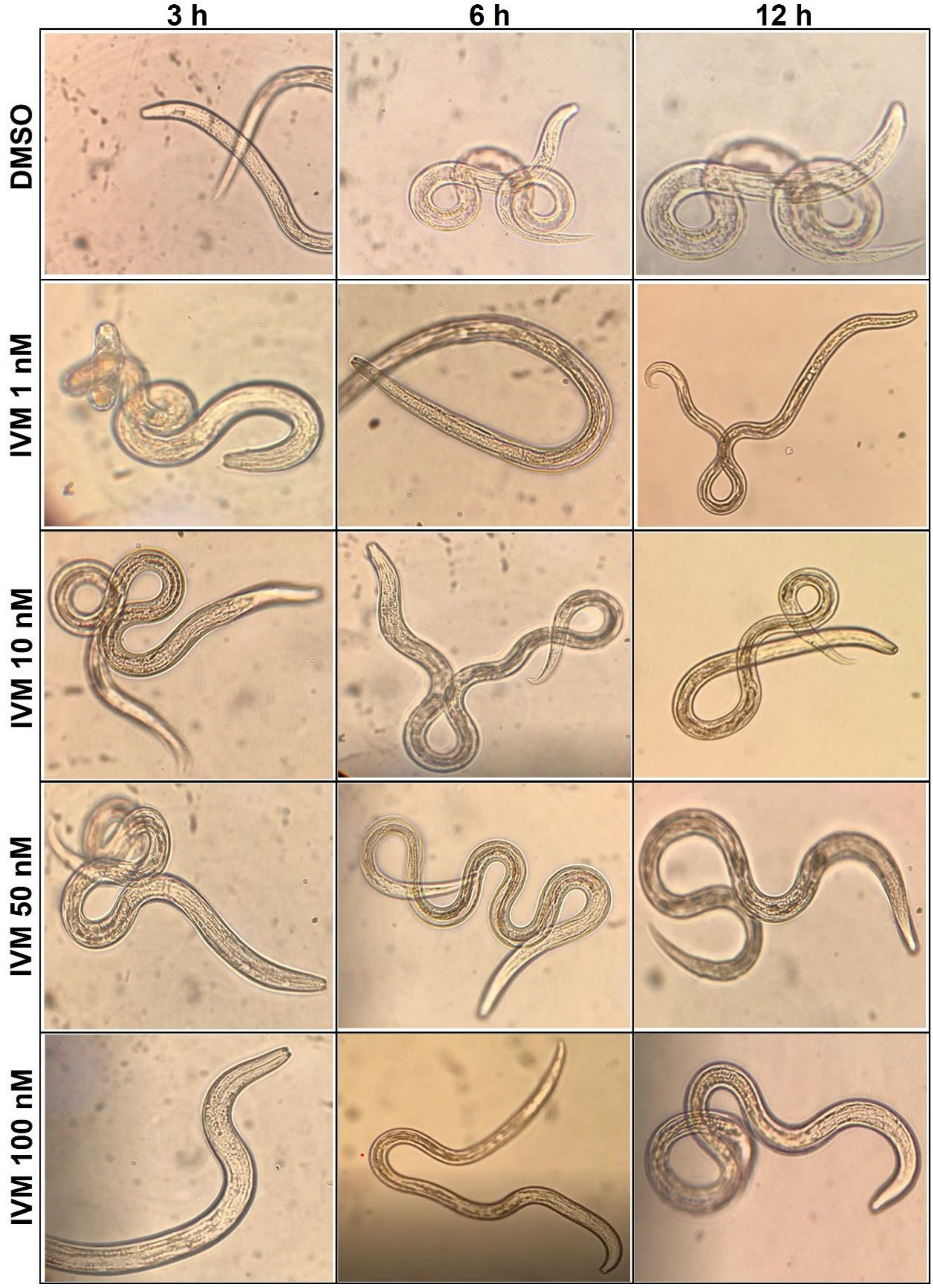

Fig. S1 *Haemonchus contortus* berlin-selected isolate (BSI) fourth-stage larvae exposed to 0.05% DMSO (vehicle control) and ivermectin (1-100 nM) for 3h, 6h, and 12h. Larvae of all conditions were assessed for visual, active pharyngeal pumping and motility. Although motility decreased with increasing ivermectin concentration, larvae were not paralyzed after 12 h of treatment.

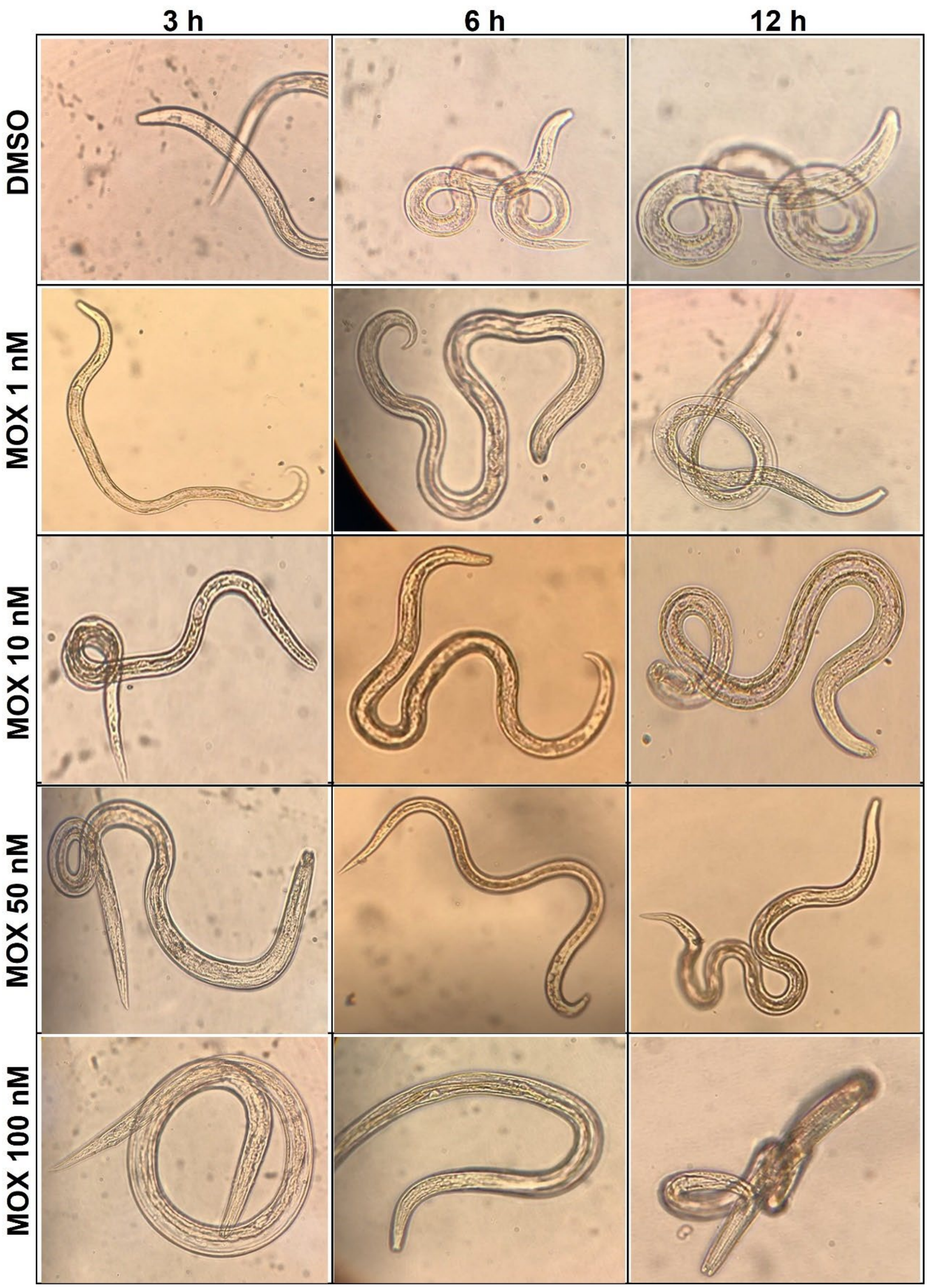

Fig. S2 *Haemonchus contortus* berlin-selected isolate (BSI) fourth-stage larvae exposed to 0.05% DMSO (vehicle control) and moxidectin (1-100 nM) for 3h, 6h, and 12h. Larvae of all conditions were assessed for visual, active pharyngeal pumping and motility. Although motility decreased with increasing moxidectin concentration, larvae were not paralyzed after 12 h of treatment.

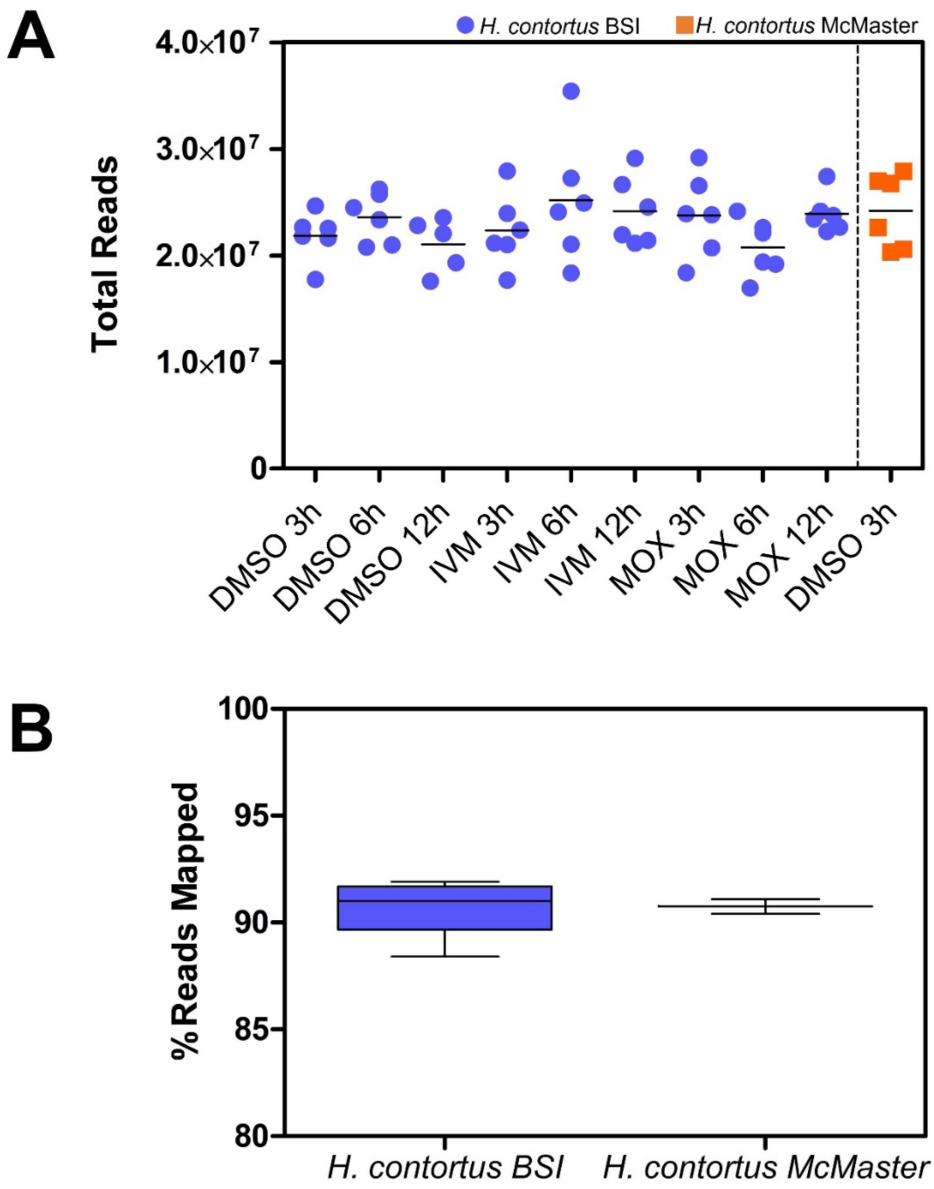

Fig. S3 A Total reads sequenced for each sample. **B** Percentage of reads mapped to MHco3 reference genome for each strain.BSI: berlin-selected isolate; IVM: ivermectin; MOX: moxidectin.

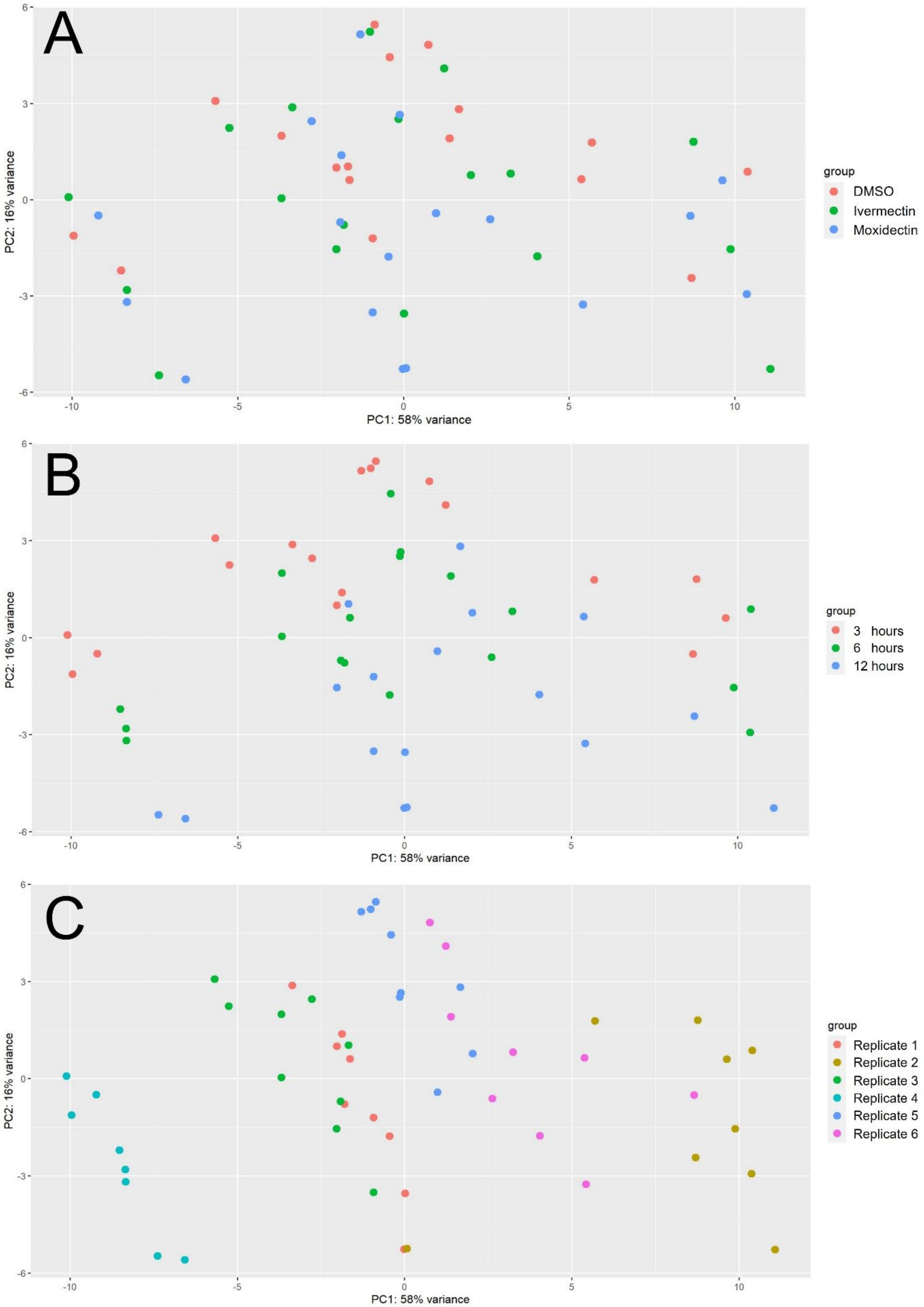

Fig. S4 Principal Component Analysis (PCA) plot illustrating sample clustering by **A** treatment group (0.05% DMSO, 100 nM ivermectin, 100 nM moxidectin), **B** incubation time (3, 6, 12 hours), and **C** replicate. PCA plot by compound and incubation time did not elucidate clustering, whereas analysis by replicate resulted in a cluster pattern attributable to the sample processing batch.

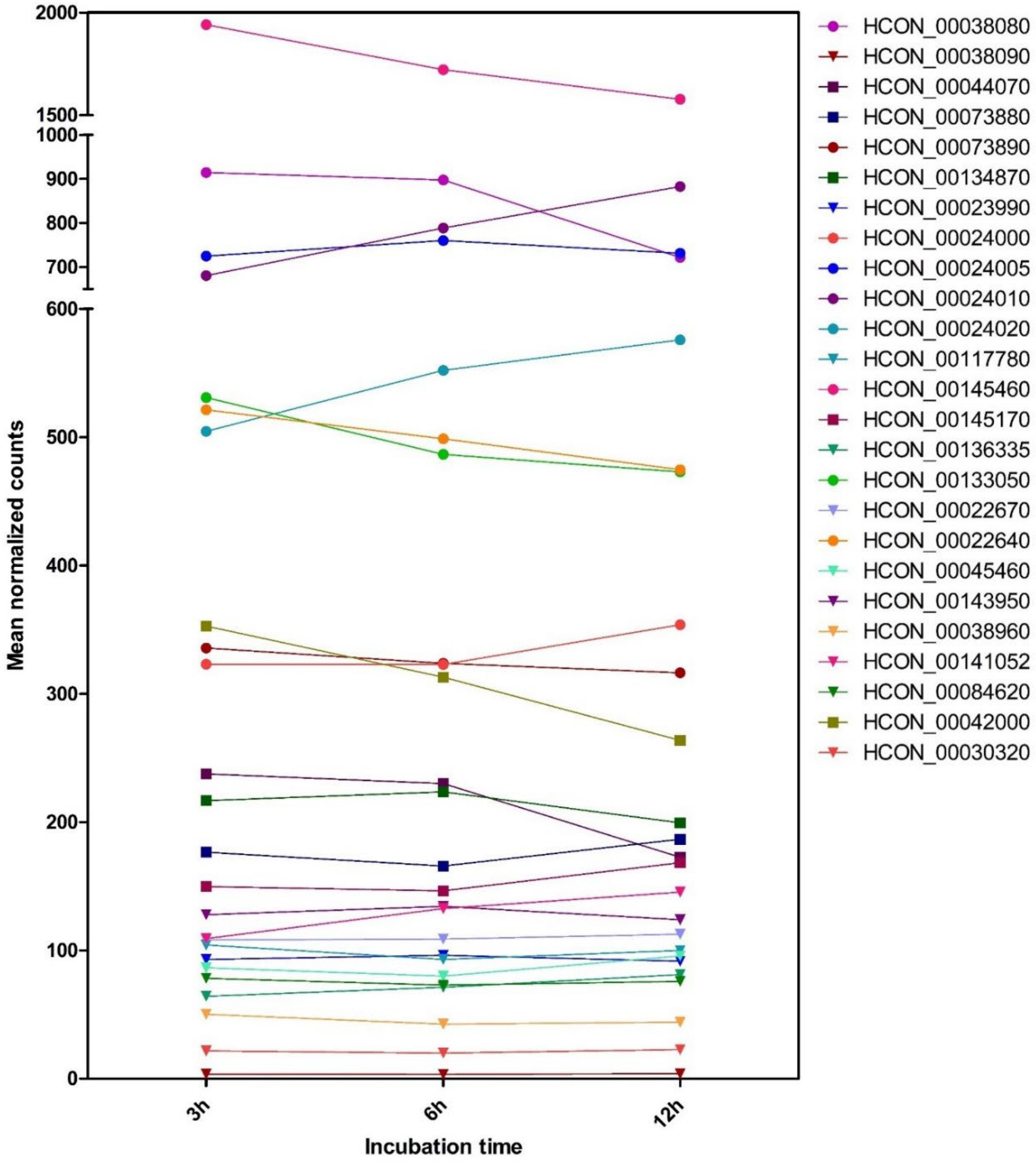

Fig. S5: Time-course of cytochrome P450 basal transcript level of the *Haemonchus contortus* Berlin selected isolate presented as mean normalized counts. Each dot represents the mean of the relative expression level from six biological replicates obtained from RNA sequencing. In order to improve readability, the SD is not shown here but the scattering of individual replicates is shown in Additional file 1: Fig. S5. Statistical analysis was conducted to compare the gene expression level of target genes between treatment groups within one isolate using the Kruskal-Wallis with Dunn's post hoc test. Gene model IDs corresponded to the *H. contortus* genome assembly PRJEB506.

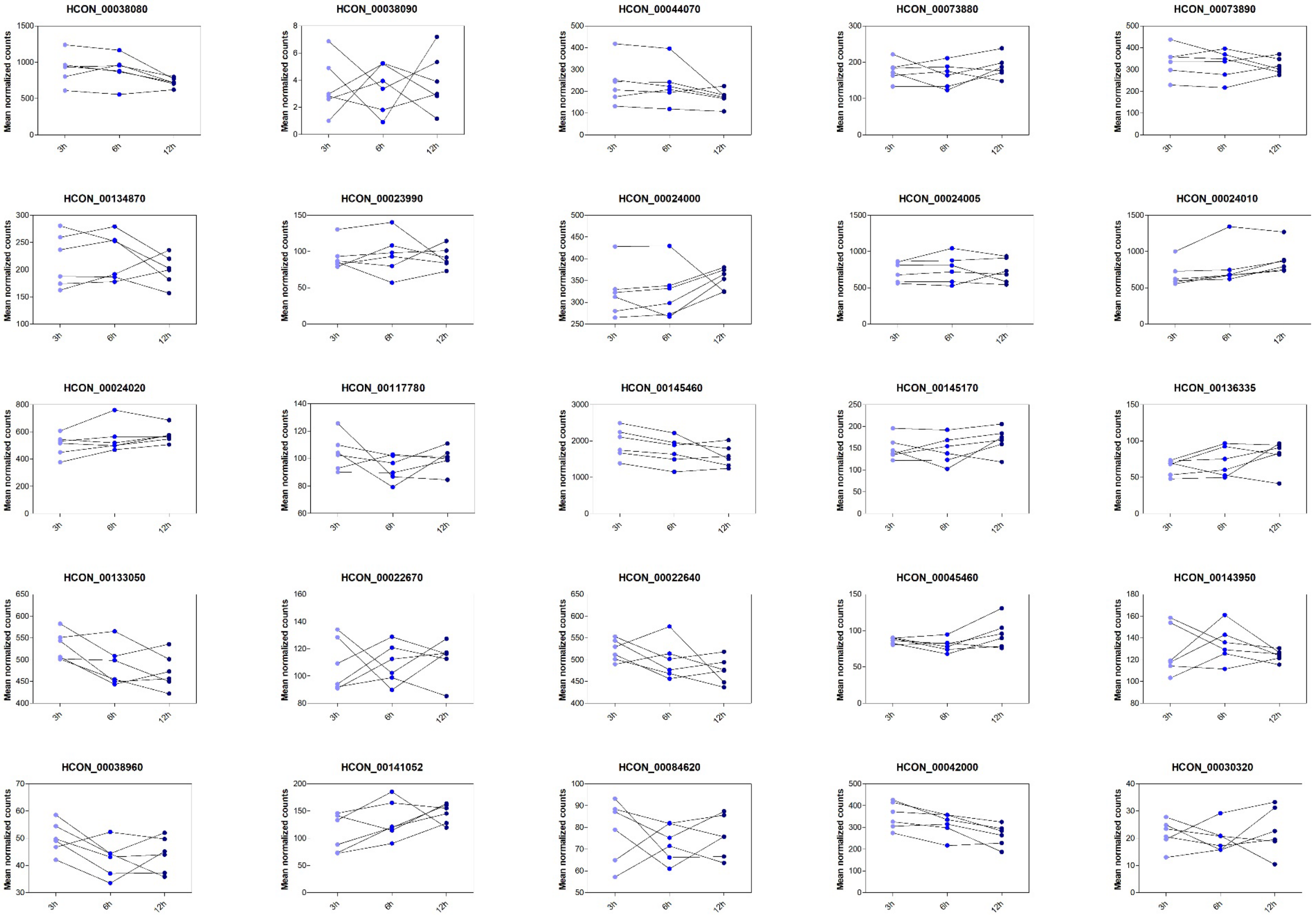
HCON_00038080
HCON_00038090
HCON_00044070
HCON_00073880
HCON_00073890
HCON_00134870
HCON_00023990
HCON_00024000
HCON_00024005
HCON_00024010
HCON_00024020
HCON_00117780
HCON_00145460
HCON_00145170
HCON_00136335
HCON_00133050
HCON_00022670
HCON_00022640
HCON_00045460
HCON_00143950
HCON_00038960
HCON_00141052
HCON_00084620
HCON_00042000
HCON_00030320
Mean normalized counts
3h
6h
12h

Fig. S6 Time course of relative basal expression (mean normalized counts) of individual cytochrome P450 transcripts in *Haemonchus contortus* berlin-selected isolate (BSI) fourth-stage larvae. The data were obtained from six biological replicates per isolate and exposed to 0.05% DMSO for 3, 6, and 12 hours. P-values were determined by Kruskal-Wallis test with Dunn's post-hoc test: ***, p-value < 0.001; **, p-value < 0.01; *, p-value < 0.05.

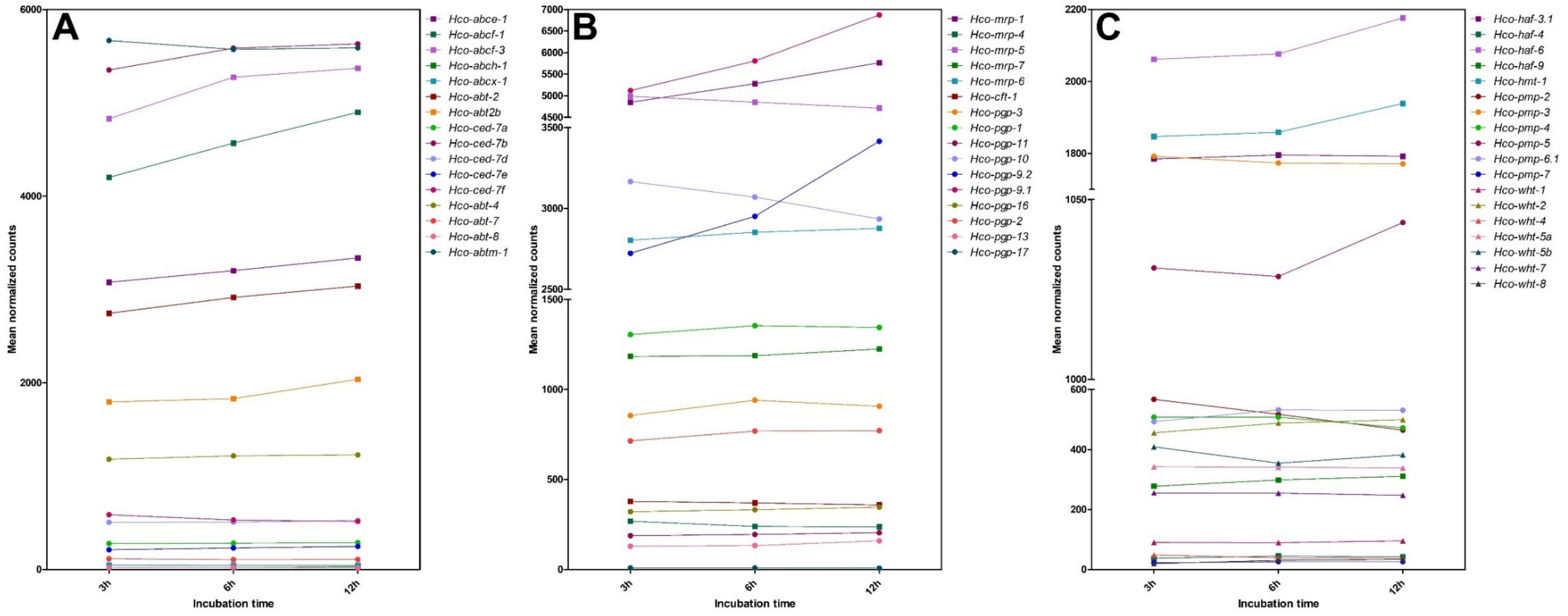

Fig. S7: Time-course of **A** *abc* and *abt,* **B** *pgp* and *mrp*, **C** *haf, hmt, pmp,* and *wht* transcripts transcript level of the *Haemonchus contortus* Berlin selected isolate (control group) presented as mean normalized counts. Each dot represents the mean of the relative expression level from six biological replicates obtained from RNA sequencing. In order to improve readability, the SD is not shown here but the scattering of individual replicates is shown in Additional file S1 Fig. S6. The Kruskal-Wallis test followed by Dunn's post-hoc test did not identify any significant changes over time. Gene model IDs correspond to the *H. contortus* genome assembly PRJEB506.

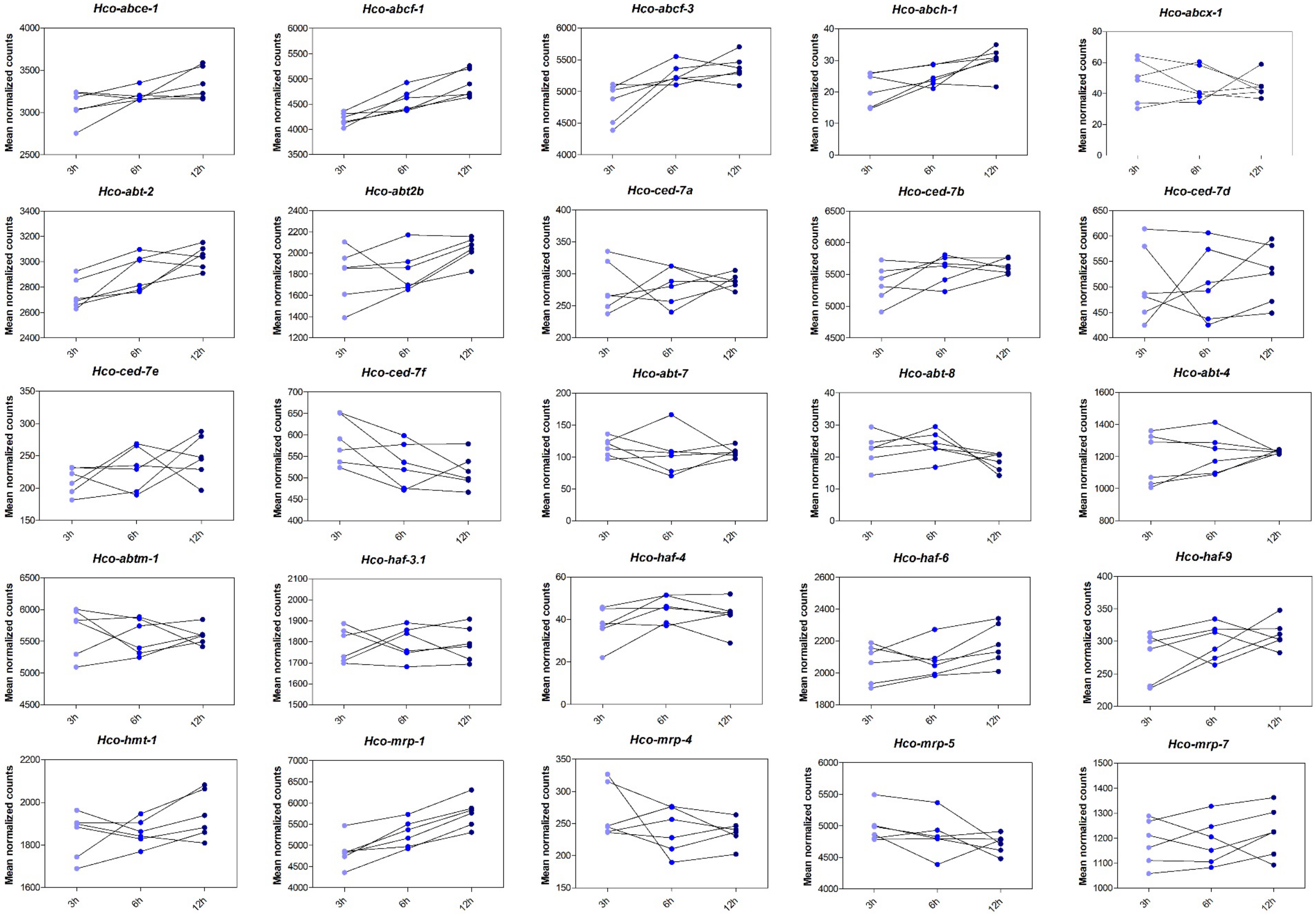

Hco-abce-1
Hco-abcf-1
Hco-abcf-3
Hco-abch-1
Hco-abcx-1
Hco-abt-2
Hco-abt2b
Hco-ced-7a
Hco-ced-7b
Hco-ced-7d
Hco-ced-7e
Hco-ced-7f
Hco-abt-7
Hco-abt-8
Hco-abt-4
Hco-abtm-1
Hco-haf-3.1
Hco-haf-4
Hco-haf-6
Hco-haf-9
Hco-hmt-1
Hco-mrp-1
Hco-mrp-4
Hco-mrp-5
Hco-mrp-7
Mean normalized counts
3h
6h
12h

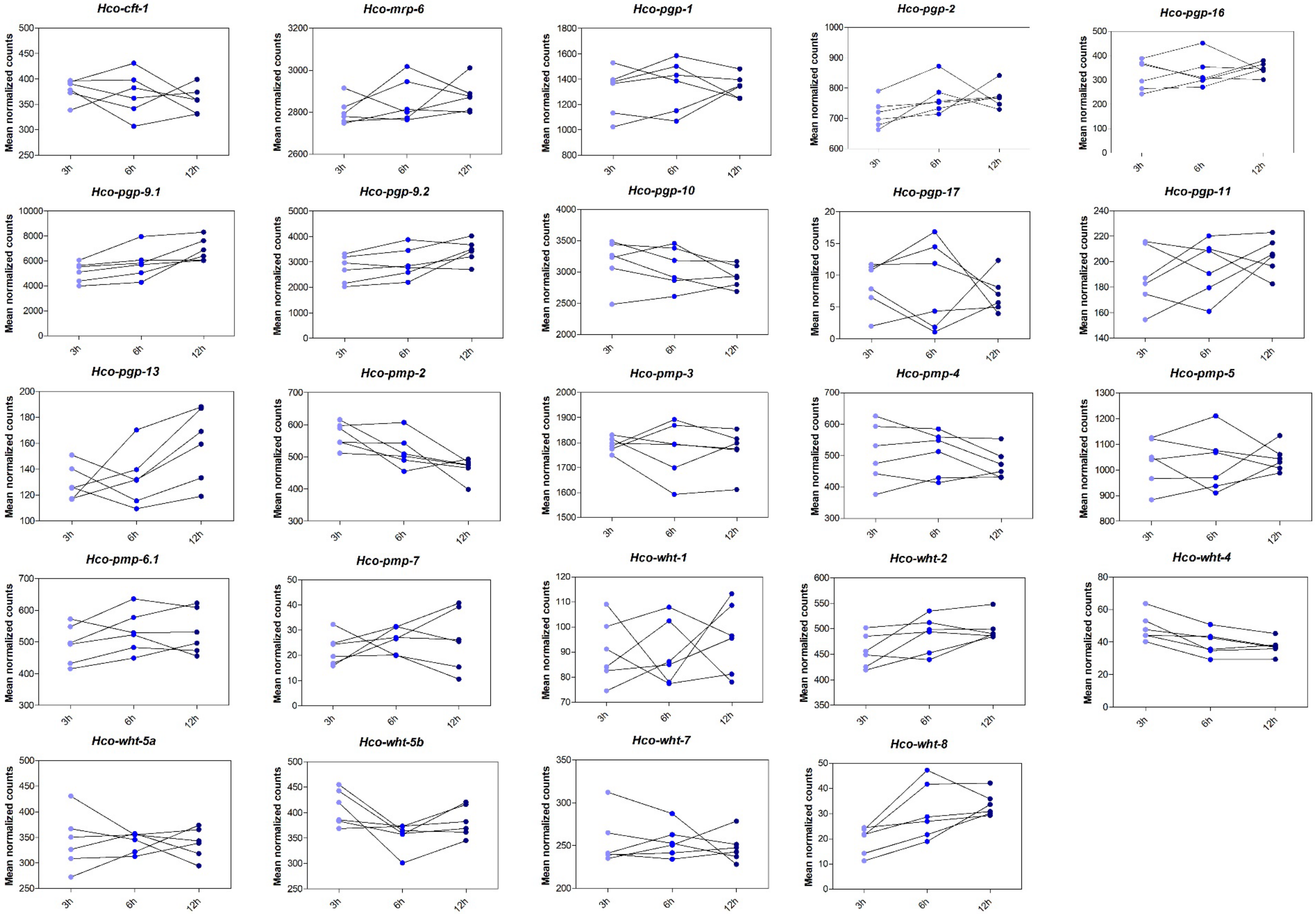
Hco-cft-1
Hco-mrp-6
Hco-pgp-1
Hco-pgp-2
Hco-pgp-16
Hco-pgp-9.1
Hco-pgp-9.2
Hco-pgp-10
Hco-pgp-17
Hco-pgp-11
Hco-pgp-13
Hco-pmp-2
Hco-pmp-3
Hco-pmp-4
Hco-pmp-5
Hco-pmp-6.1
Hco-pmp-7
Hco-wht-1
Hco-wht-2
Hco-wht-4
Hco-wht-5a
Hco-wht-5b
Hco-wht-7
Hco-wht-8
Mean normalized counts
3h
6h
12h

Fig. S8 Time course of relative expression (mean normalized counts) of ABC transporter transcripts in *Haemonchus contortus* berlin-selected isolate (BSI) fourth-stage larvae. The data were obtained from six biological replicates per isolate and exposed to 0.05% DMSO for 3, 6, and 12 hours. P-values were determined by Kruskal-Wallis test with Dunn's post-hoc test: ***, p-value < 0.001; **, p-value < 0.01; *, p-value < 0.05. *abc*: **AB**C transporter **C**lass (including ABC transporter class E-H, and ABC Transporter eXtended); *abt*: **AB**C **T**ransporter family; *abtm*: **AB**C **T**ransporter **M**itochondrial; *haf*: **HA**l**F** transporter; *hmt*: **H**eavy **M**etal **T**olerance factor; *pmp*: **P**eroxisomal **M**embrane **P**rotein related; *wht*: **WH**i**T**e *Drosophila* related ABC transporter.

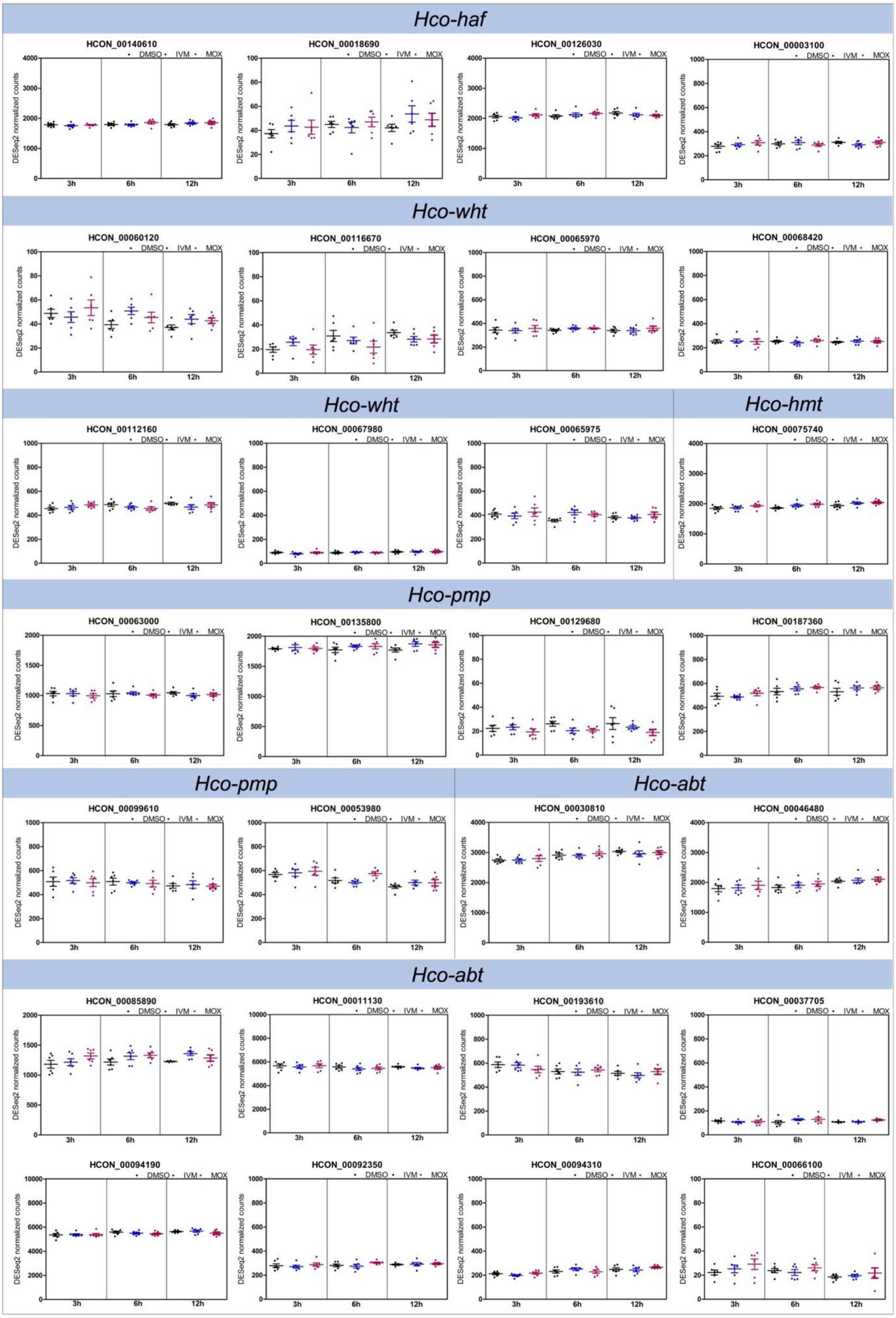
Hco-haf
HCON_00140610
HCON_00018690
HCON_00126030
HCON_00003100
Hco-wht
HCON_00060120
HCON_00116670
HCON_00065970
HCON_00068420
Hco-wht
HCON_00112160
HCON_00067980
HCON_00065975
Hco-hmt
HCON_00075740
Hco-pmp
HCON_00063000
HCON_00135800
HCON_00129680
HCON_00187360
Hco-pmp
HCON_00099610
HCON_00053980
Hco-abt
HCON_00030810
HCON_00046480
Hco-abt
HCON_00085890
HCON_00011130
HCON_00193610
HCON_00037705
HCON_00094190
HCON_00092350
HCON_00094310
HCON_00066100
DMSO
IVM
MOX
DESeq2 normalized counts
3h
6h
12h

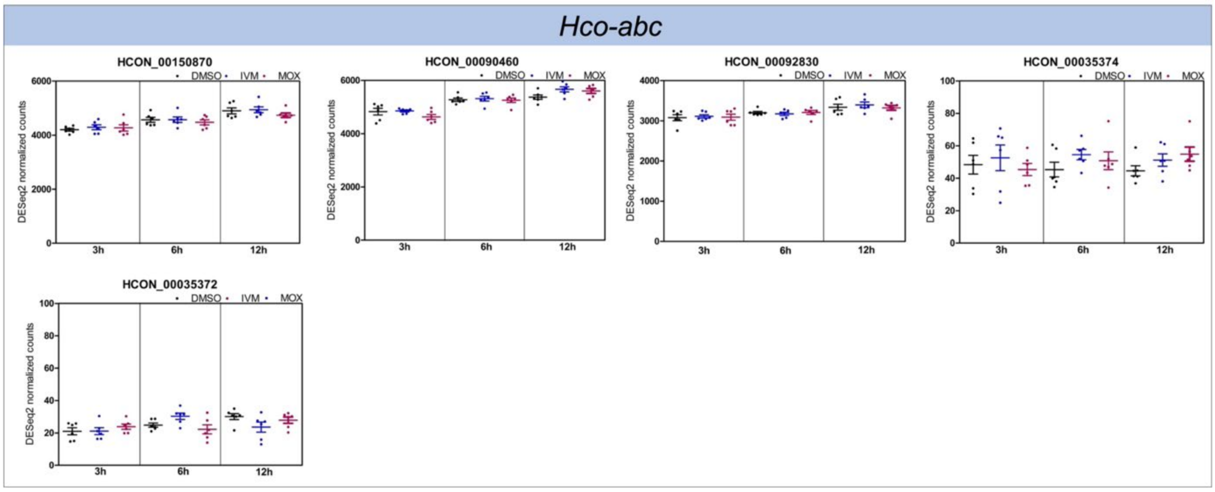

Fig. S9 Time-course differential gene expression analysis of *Haemonchus contortus* ABC transporter based on RNA sequencing. Scatter dot plot showing mean normalized counts (DESeq2) of 100 nM ivermectin, 100 nM moxidectin, and 0.05% DMSO-treated *H. contortus* berlin-selected isolate (BSI) fourth stage larvae for 3h, 6h, and 12h. Data were obtained from six biological replicates. Statistical analysis to compare the gene expression level of target genes between treatment groups (factor 1) and over time (factor 2) was carried out using a two-way ANOVA followed by a Bonferroni post hoc test: ***, p-value < 0.001; **, p-value < 0.01; *, p-value < 0.05. *H. contortus* ABC transporter genes are grouped into gene families, according to Mate et al. (2022). Gene model IDs (HCON) correspond to the *Haemonchus contortus* genome assembly PRJEB506 (50). *abc*: **AB**C transporter **C**lass (including ABC transporter class E-H, and ABC Transporter eXtended); *abt*: **AB**C **T**ransporter family; *abtm*: **AB**C **T**ransporter **M**itochondrial; *haf*: **HA**l**F** transporter; *hmt*: **H**eavy **M**etal **T**olerance factor; *pmp*: **P**eroxisomal **M**embrane **P**rotein related; *wht*: **WH**i**T**e *Drosophila* related ABC transporter.
